# Fabrication and Characterization of Chitosan-Integrated Polyvinyl Alcohol–Polycaprolactone/Tea Tree Oil Nanofibrous Scaffolds via Dual-Nozzle Electrospinning for Wound Dressing Applications

**DOI:** 10.3390/polym18202558

**Published:** 2026-10-17

**Authors:** Natavan Ismayilova, Tubanur Avci, Zekiye Akdag, Ridvan Yildirim, Zehra Kanli, Ahmet Akif Kızılkurtlu, Oguzhan Gunduz, Canan Dogan

**Affiliations:** 1 Chemical Engineering Department, Engineering Faculty, Istanbul University Cerrahpasa, Istanbul 34320, Turkey; 2 Department of Metallurgical and Materials Engineering, Faculty of Technology, Marmara University, Istanbul 34722, Turkey; 3 Department of Biophysics, Faculty of Medicine, Istanbul Arel University, Istanbul 34010, Turkey; 4 Department of Biomedical Engineering, Faculty of Engineering and Natural Sciences, Istanbul Atlas University, Istanbul 34403, Turkey; 5 Atlas University Research Center (ARC), Istanbul Atlas University, Istanbul 34403, Turkey

**Keywords:** dual-nozzle co-electrospinning, hybrid nanofibrous mats, tea tree oil, wound dressing

## Abstract

Hybrid nanofibrous mats comprising distinct hydrophilic and hydrophobic fiber populations were fabricated by simultaneous dual-nozzle co-electrospinning for potential wound-dressing applications. Four formulations—polyvinyl alcohol (PVA)–polycaprolactone (PCL), PVA/Chitosan (CS)–PCL, PVA-PCL/Tea Tree Oil (TTO), and PVA/CS-PCL/TTO—were systematically evaluated in terms of their morphological, physicochemical, mechanical, release, and in vitro cellular properties. All formulations exhibited continuous, bead-free fiber morphologies. FTIR and DSC analyses indicated the physical incorporation of CS and TTO and composition-dependent changes in polymer-chain organization. TTO incorporation improved surface wettability, decreasing the water contact angle to 46.69 ± 2.80° and 57.05 ± 1.40° in the CS-free and CS-containing formulations, respectively. The PVA/CS-PCL/TTO mat exhibited the highest tensile strength (19.17 ± 3.06 MPa) and strain at break (10.82 ± 1.81%). Cumulative TTO release reached 97.63–99.75% within 8 h. MTT analysis showed favorable HDF metabolic activity, with the highest response observed for PVA/CS-PCL/TTO on day 7. Overall, the results support the further investigation of these hybrid nanofibrous mats as potential wound-dressing platforms.

## 1. Introduction

Chronic and non-healing wounds remain a major clinical challenge, creating a continued need for advanced wound-care materials that can provide a favorable environment for tissue repair [1,2,3]. In this context, electrospun nanofibrous mats have emerged as promising candidates for wound dressing applications because their fibrous architecture can mimic key structural features of the native extracellular matrix (ECM) [1,2,3]. Their high surface-area-to-volume ratio and interconnected porous structure provide favorable conditions for cell–material interactions and mass transport at the wound interface [1,2]. Moreover, the versatility of electrospinning enables the incorporation of bioactive compounds into fibrous matrices, allowing the physicochemical and biological properties of wound dressing materials to be tailored [2,3]. Collectively, these characteristics support the use of electrospun nanofibers as versatile platforms for developing functional wound dressings.

A recurring challenge in the development of multicomponent electrospun materials is the integration of constituents with markedly different physicochemical and processing characteristics. Combining natural and synthetic polymers within hybrid fibrous constructs can provide complementary properties that are difficult to achieve using a single polymer system [4,5,6]. In this regard, multi-nozzle co-electrospinning offers a versatile strategy for processing distinct polymer formulations independently while integrating the resulting fibers within a common fibrous construct. Shahverdi et al. fabricated hybrid CS/PVA-PCL nanofibers using a two-nozzle electrospinning system, while Ranjbar-Mohammadi et al. employed double-nozzle electrospinning to combine PVA/CS and gelatin/PCL fiber systems for diabetic wound-healing applications [4,5]. These studies demonstrate the feasibility of integrating distinct polymer formulations through simultaneous electrospinning. Building on this, recent advances in dual-polymer electrospinning have highlighted the advantage of multi-nozzle configurations in processing incompatible polymer solutions independently, thereby preventing thermodynamic phase separation while tailoring the structural and mechanical properties of hybrid matrices [7]. In the present study, this approach is particularly relevant because the hydrophilic PVA-based phase, optionally containing chitosan (CS), and the comparatively hydrophobic PCL-based phase, optionally loaded with tea tree oil (TTO), possess distinct solution and processing characteristics. Processing these formulations through separate nozzles enables their independent preparation and simultaneous co-deposition, allowing compositionally distinct fibers to be integrated within a single hybrid nanofibrous mat.

Poly(vinyl alcohol) (PVA), chitosan (CS), and poly(ε-caprolactone) (PCL) possess complementary properties that make their combination attractive for electrospun wound dressing applications [3,6,8,9,10]. PVA is a hydrophilic, biocompatible, and readily electrospinnable polymer that has been extensively investigated for skin wound dressings [8,9]. However, its pronounced interaction with water can compromise the dimensional and structural stability of PVA-based fibrous constructs under aqueous conditions, thereby motivating its combination or modification with other polymers [8]. CS is a naturally derived polysaccharide widely investigated in wound-related biomaterials because of its biocompatibility, biodegradability, and biological functionality [3,9]. Nevertheless, electrospinning CS alone is challenging, and blending with readily fiber-forming polymers such as PVA is commonly conducted to improve processability [3,9]. In contrast, PCL is a biodegradable polyester with favorable electrospinnability and structural stability, although its intrinsic hydrophobicity and comparatively slow degradation may limit its biological performance in wound-related environments [6,10,11]. Hybridization of PVA/CS- and PCL-based fibers therefore provides a rational approach to combining the hydrophilic and biologically favorable properties of PVA/CS with the structural stability and slower degradation behavior of PCL [4,5,6].

TTO, an essential oil derived from Melaleuca alternifolia, has been investigated as a natural bioactive component for the functionalization of electrospun biomaterials [12,13,14,15]. Although TTO is widely recognized for its antimicrobial activity, studies have also reported antioxidant, anti-inflammatory, and wound-related biological effects [12,15]. Notably, Doustdar et al. incorporated TTO into an electrospun PCL/soy protein isolate mat and reported enhanced antioxidant activity, favorable cytocompatibility, and accelerated in vitro wound closure following TTO incorporation [12]. However, the hydrophobic nature and low aqueous solubility of TTO can complicate its incorporation into hydrophilic polymer systems. Accordingly, several strategies have been investigated to integrate TTO into electrospun fibers, including direct incorporation, post-fabrication absorption, reduced-pressure processing, and emulsion-based encapsulation [13,14,15]. For example, Jiang et al. employed Pickering-emulsion electrospinning to incorporate TTO into a hydrophilic chitosan/PVA-based nanofibrous matrix [15]. In addition, Zhou et al. reported that a TTO nanoemulsion-loaded hydrogel promoted fibroblast migration and angiogenesis and reduced α-smooth muscle actin (α-SMA) expression, suggesting its potential to support wound healing while limiting scar formation [1]. In the present study, TTO was instead incorporated into the PCL-based spinning phase, enabling the TTO-containing PCL formulation and the aqueous PVA or PVA/CS formulation to be prepared and processed independently.

Despite considerable progress in electrospun wound dressing systems, limited attention has been given to systematic dual-nozzle designs that combine compositionally distinct hydrophilic and hydrophobic fibers while selectively incorporating TTO into one of the fiber phases. PVA/CS-PCL-containing hybrid systems have previously been fabricated using multi-nozzle electrospinning, and PVA-PCL-based multilayer systems containing CS have also been investigated for wound dressing applications [4,5,6]. In parallel, TTO has been incorporated into PCL-containing, PVA/CS-based, PHBV, and other electrospun polymer systems [12,13,14,15]. However, these studies do not systematically compare a dual-nozzle PVA(/CS)-PCL(/TTO) platform designed to distinguish the individual contributions of CS and TTO, as well as their combined effects within the same hybrid architecture. Such a comparative design may provide further insight into how the composition of independently electrospun fibers influences the structural, physicochemical, mechanical, and cellular properties of hybrid nanofibrous materials.

Therefore, the present study aimed to develop and systematically evaluate hybrid nanofibrous mats fabricated by simultaneous dual-nozzle co-electrospinning, combining PVA- or PVA/CS-based fibers with PCL- or TTO-loaded PCL fibers. Four formulations—PVA-PCL, PVA/CS-PCL, PVA-PCL/TTO, and PVA/CS-PCL/TTO—were designed to elucidate the individual and combined effects of CS and TTO on the resulting fibrous constructs. The developed nanofibrous mats were comparatively evaluated in terms of their morphological, thermal, mechanical, swelling, degradation-related, and in vitro cellular characteristics. Particular emphasis was placed on determining how TTO incorporation into the PCL fiber phase, either alone or in combination with CS in the PVA-based phase, modulates the overall performance of the hybrid constructs. Through this comparative design, the study seeks to establish composition–structure–property relationships and assess the potential of these co-electrospun hybrid nanofibrous mats as cytocompatible platforms for prospective wound dressing applications. Additionally, the effects of the developed hybrid platforms on HDF metabolic activity were investigated using the MTT assay to provide a preliminary assessment of their in vitro cellular response and cytocompatibility.

## 2. Materials and Methods

### 2.1. Materials

PCL (Mn = 80,000 g mol^−1^) and Chitosan (CS) (Mw = 100,000 g mol^−1^) were obtained from Sigma-Aldrich (St. Louis, MO, USA). PVA (Mw = 89,000–98,000 g mol^−1^, 99% hydrolyzed) was purchased from Sigma-Aldrich (Gillingham, UK). Tween^®^ 80, used as a nonionic surfactant, was purchased from Merck KGaA (Darmstadt, Germany). Tea Tree Oil (TTO), a 100% pure essential oil derived from *Melaleuca alternifolia*, was obtained from NU-KA Defne Essencia (Alanya, Antalya, Türkiye).Glacial acetic acid (AA, Mw = 60.05 g mol^−1^, ≥100%) was purchased from ISOLAB (Windsor, ON, Canada), and chloroform (CHCl_3_) was obtained from Sigma-Aldrich (St. Louis, MO, USA).

### 2.2. Methods

#### 2.2.1. Preparation of the Spinning Formulations

The PCL/TTO spinning formulation was prepared by dissolving PCL in chloroform at a concentration of 10% (*w*/*v*) under magnetic stirring at room temperature. After complete dissolution of PCL for 1 h, TTO was added at 10% (*v*/*v*), and the resulting formulation was stirred for an additional 1 h at room temperature.

Separately, a 13% (*w*/*v*) PVA solution was prepared by dissolving PVA in distilled water under continuous magnetic stirring at 80–90 °C for 1 h until a homogeneous solution was obtained. A 3% (*w*/*v*) CS stock solution was prepared separately in an acetic acid/deionized water mixture (9:1, *v*/*v*) before blending with the aqueous PVA solution. The prepared PVA and CS solutions were subsequently combined at a PVA/CS volume ratio of 7:3 and mixed until homogeneous. The resulting PVA/CS blend had a nominal acetic acid/water solvent ratio of 27:73 (*v*/*v*). The solvent systems, polymer concentrations, and PVA/CS blending ratio were selected based on preliminary electrospinning trials to obtain formulations suitable for fiber production under the processing conditions employed. Tween^®^ 80 was added to both spinning formulations at a concentration of 3% (*v*/*v*), followed by magnetic stirring for 15 min before electrospinning. The polymer and additive concentrations reported in Table 1 are based on the final volume of each spinning formulation after TTO and Tween 80 incorporation, where applicable.

#### 2.2.2. Simultaneous Dual-Nozzle Co-Electrospinning Processes

**Figure 1:**
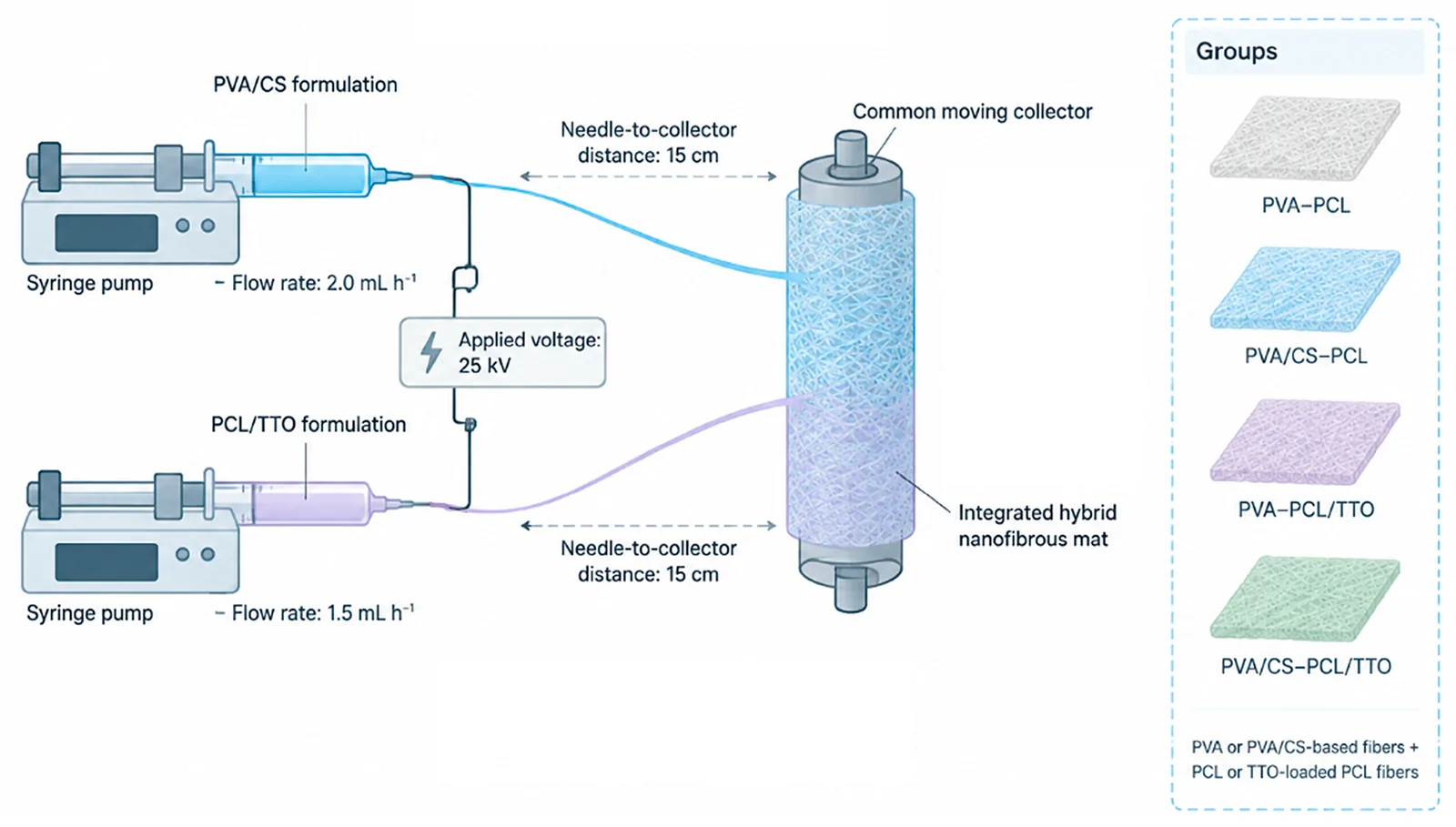
Schematic representation of the overall fabrication process.

The hybrid nanofibrous mats were fabricated using a simultaneous dual-nozzle co-electrospinning method. The overall fabrication process is schematically illustrated in Figure 1.

Simultaneous dual-nozzle co-electrospinning was performed at room temperature of 23–25 °C and an approximate relative humidity of 40%. The PCL/TTO and PVA/CS formulations were loaded into separate syringes and independently electrospun through two individual needles. The PCL/TTO formulation was delivered at a flow rate of 1.5 mL h^−1^, whereas the PVA/CS formulation was delivered at 2.0 mL h^−1^. An applied voltage of 25 kV was used for both formulations and the needle-to-collector distance was set at 15 cm for each nozzle. The independently generated fibers were concurrently deposited onto a common moving collector that rotated while simultaneously undergoing lateral reciprocating motion, resulting in integrated hybrid nanofibrous mats.

### 2.3. Scanning Electron Microscopy (SEM) Analysis

A scanning electron microscope (SEM; EVO 10, Zeiss, Oberkochen, Germany) was used to analyze the morphology of the generated nanofibers. To avoid electrostatic charge and enhance image quality, the samples were sputtered with a thin coating of gold/palladium at 18 mA under vacuum for 120 s before imaging. Using ImageJ software (v1.47, NIH, Bethesda, MD, USA), fiber diameters were determined from SEM images. For statistical accuracy, at least 100 measurements were made from various parts of each sample. The fiber diameter distributions were evaluated collectively for the hybrid mats, without differentiating between the individual polymer fiber populations.

### 2.4. Chemical Analysis of the Nanofibrous Mats

The chemical composition and functional groups of the nanofibrous mats were characterized by Fourier transform infrared (FTIR) spectroscopy using an FT/IR-4700 spectrometer (JASCO Corporation, Tokyo, Japan). Measurements were performed in the 4000–400 cm^−1^ region with a resolution of 4 cm^−1^, and 32 scans were acquired for each sample.

### 2.5. Differential Scanning Calorimetry (DSC)

Differential scanning calorimetry (DSC) was employed to characterize the thermal behavior of the nanofibrous mats using a DSC-60 Plus calorimeter (Shimadzu Corporation, Kyoto, Japan). For each measurement, approximately 5 mg of sample was enclosed in a standard aluminum pan and subjected to heating from 25 to 300 °C at 20 °C min^−1^. Thermal transitions were subsequently evaluated from the obtained DSC curves.

### 2.6. Mechanical Testing

The tensile performance of the nanofibrous mats was assessed using a Shimadzu EZ-LX universal testing machine (Shimadzu Corporation, Kyoto, Japan). Rectangular specimens measuring 10 mm × 50 mm were tested at a crosshead speed of 5 mm min^−1^ after applying a preload of 0.1 N. Three specimens from each group were analyzed, and the resulting data were used to determine tensile stress and strain characteristics. Rectangular specimens (approximately 10 mm × 50 mm) were prepared, and the precise width and thickness of each individual specimen were recorded across 3–5 distinct locations using a digital micrometer prior to testing to ensure accurate cross-sectional area calculations. All samples were fabricated in unified electrospinning batches under controlled environmental conditions (23–25 °C and ~40% RH) to minimize batch-to-batch variation.

### 2.7. Swelling and Degradation Measurements

To evaluate the fluid absorption capacity and structural stability of the produced nanofibrous patches, swelling and degradation tests were performed in phosphate-buffered saline (PBS, pH 7.4). Three approximately equal-weighted specimens from each nanofiber group (*n* = 3) were prepared in order to evaluate these characteristics, and their initial dry mass (W_0_) was noted. Subsequently, 1 mL of PBS was added to 1.5 mL microcentrifuge tubes containing the samples. In order to simulate physiological conditions, the produced samples were incubated at 37 °C in a thermal shaker (BIOSAN TS-100). The samples were taken out of the solution at regular intervals of 24 h to track the swelling kinetics over a prolonged period of time. Each sample’s swollen mass (W_w_) was measured immediately after the excess liquid on the nanofiber surfaces was carefully blotted with filter paper. This procedure was repeated at regular intervals for up to 14 days. Equation (1) was used to calculate the swelling ratio (S):

(1) $$S=\frac{W_{w}-W_{0}}{W_{0}}\times 100$$

To evaluate the structural stability of the nanofibrous patches, a degradation test was performed over a total period of 7 days. The patches were cut into equal pieces, weighed (*W*_i_), and incubated in 1 mL of PBS at 37 °C for 24 h in an Eppendorf thermal shaker. Following each designated incubation period, the PBS was removed, and the nanofiber samples were transferred back into empty Eppendorf tubes. The tubes were then dried at 37 °C for 24 h with their lids open to ensure complete dryness. After drying, the final weights (*W*_f_) of the nanofibers were recorded, and the degradation percentage (D) was calculated using Equation (2):

(2) $$D=\frac{W_{i}-W_{f}}{W_{i}}\times 100$$

### 2.8. In Vitro Drug Release Study

The in vitro release testing of drug-loaded nanofiber patches (PVA-PCL/TTO and PVA/CS-PCL/TTO) was conducted in a PBS solution at pH 7.4 using a thermal shaker (BIOSAN-TS 100, Riga, Latvia) at 37 °C and 300 rpm. A linear calibration curve for TTO at different concentrations (40, 20, 10, 5, and 2.5 ppm) was determined in the 190–300 nm wavelength range via UV spectrophotometry (Shimadzu UV-3700, Kyoto, Japan). Drug-loaded nanofiber patches, each weighing 5 mg, were transferred to an Eppendorf tube containing 1 mL of PBS. To determine the amount of TTO released from the nanofibers, absorbance values at 265 nm were recorded using a UV spectrophotometer. After each measurement taken at predetermined time intervals (0.25, 0.5, 1, 2, 3, 4, 6, 8, 12, 24, and 48 h), the 1 mL of PBS was replaced with fresh PBS to minimize the accumulation of TTO in the release medium. Cumulative release was calculated as the sum of the amounts measured in each completely replaced aliquot and expressed relative to the total amount released at 48 h (C_t_/C_∞_). All measurements were repeated 3 times during the experiments.

To investigate the release kinetics of TTO from nanofiber mats, four kinetic models (first-order, Higuchi, Korsmeyer–Peppas, and Peppas–Sahlin) were considered, as shown in Equations (3)–(6) [16,17].

(3) $$\frac{C_{t}}{C_{\infty }}=1-e^{-k_{1}t}$$

(4) $$\frac{C_{t}}{C_{\infty }}=k_{h}t^{1/2}$$

(5) $$\frac{C_{t}}{C_{\infty }}=k_{p}t^{n}$$

(6) $$\frac{C_{t}}{C_{\infty }}=k_{d}t^{m}+k_{r}t^{2m}$$

In the equations above, *C_t_* represents the amount of drug (TTO) released, *C_∞_* represents the maximum amount of TTO that can be released, and *k*_1_, *k_h_*, *k_p_*, *k_d_*, and *k_r_* are the kinetic constants of the first-order, Higuchi, Korsmeyer–Peppas, and Peppas–Sahlin models, respectively. The “*n*” diffusion exponent in the Korsmeyer–Peppas model indicates the drug release mechanism. The “*m*” exponent in the Peppas–Sahlin model represents the geometry-dependent Fickian diffusion exponent.

### 2.9. Water Contact Angle Measurements

The surface wettability of the co-electrospun hybrid nanofibrous mats was evaluated via static water contact angle (WCA) measurements using an OCA 15EC goniometer (DataPhysics, Filderstadt, Germany). Prior to testing, representative specimens (approximately 10 mm × 10 mm) were carefully cut from the co-deposited nanofibrous mats and stored under dry conditions. Deionized water droplets (3 μL) were gently placed onto the flat surface of the nanofibrous mats, and analysis were performed at ambient temperature and humidity. To ensure statistical reliability, contact angle values were averaged from four replicate measurements taken at distinct locations across each sample, with results expressed as the mean ± standard deviation.

### 2.10. Rheological Measurements

The rheological properties of the electrospinning solutions were characterized using an MCR 302e rotational rheometer (Anton Paar, Graz, Austria) equipped with a 25 mm parallel-plate geometry at a fixed measuring gap of 1 mm. All measurements were performed at 25 °C to evaluate the flow behavior and viscoelastic properties of the formulations. Steady-shear rotational tests were conducted over a shear rate range of 1 to 100 s^−1^ to determine apparent viscosity and assess the flow behavior of the solutions. Prior to the steady-shear measurements, the samples were allowed to equilibrate at the measurement temperature for 1 min. Dynamic oscillatory amplitude sweep tests were carried out at a constant frequency of 1 Hz, varying the strain amplitude from 0.01% to 100% (over 25 data points) to determine the storage modulus (G′) and loss modulus (G″). The samples were allowed to recover their structure for at least 3 min before the amplitude sweep tests.

### 2.11. In Vitro Cytotoxicity and Cell Viability Assay

#### 2.11.1. Cell Viability Analysis of HDF Cells on Tea Tree Oil Nanofibers

Human dermal fibroblast (HDF) cells (PCS-201-012™, ATCC, Manassas, VA, USA) were employed to evaluate the cell viability of electrospun tea tree oil nanofibers. The cells were cultured in Dulbecco’s Modified Eagle Medium (DMEM, Gibco, Grand Island, NY, USA) supplemented with 10% fetal bovine serum (FBS, Gibco, USA) and 1% penicillin–streptomycin (Gibco, USA). Cultures were maintained at 37 °C in a humidified atmosphere containing 5% CO_2_. Prior to cell seeding, all nanofiber samples were sterilized under ultraviolet (UV) irradiation for 30 min. Cell viability on the tea tree oil-loaded nanofibers was quantitatively evaluated using the MTT Cell Proliferation and Cytotoxicity Assay Kit (Cat. No: E-CK-A341, Elabscience Biotechnology Inc., Houston, TX, USA) according to the manufacturer’s instructions. Briefly, HDF cells were seeded onto sterilized nanofiber mats and incubated for 1, 3, and 7 days. At each designated time point, the culture medium was aspirated and replaced with 100 µL of fresh DMEM containing 50 µL of MTT reagent, followed by a 2.5-h incubation at 37 °C to allow the formation of purple formazan crystals by mitochondrial dehydrogenases. To dissolve the crystals, dimethyl sulfoxide (DMSO; Sigma-Aldrich, St. Louis, MO, USA) was added, and the samples were further incubated at 37 °C for 10 min [18,19,20]. The absorbance was subsequently measured at 570 nm using a microplate reader (Thermo Multiskan SkyHigh, Waltham, MA, USA). The PVA-PCL formulation, which did not contain CS or TTO, was used as the reference control for comparison of the cellular responses among the nanofibrous formulations. MTT responses of the PVA/CS-PCL, PVA-PCL/TTO, and PVA/CS-PCL/TTO groups were evaluated relative to the PVA-PCL control at the corresponding time point.

#### 2.11.2. Statistical Analysis

All experiments were conducted in triplicate (*n* = 3). Data were expressed as the mean ± standard error of the mean (SEM). Statistical significance was determined using two-way analysis of variance (ANOVA) followed by Tukey’s multiple comparison test, with a *p*-value of <0.05 considered statistically significant.

## 3. Results and Discussions

### 3.1. Morphological Analysis of the Nanofibrous Mats

**Figure 2:**
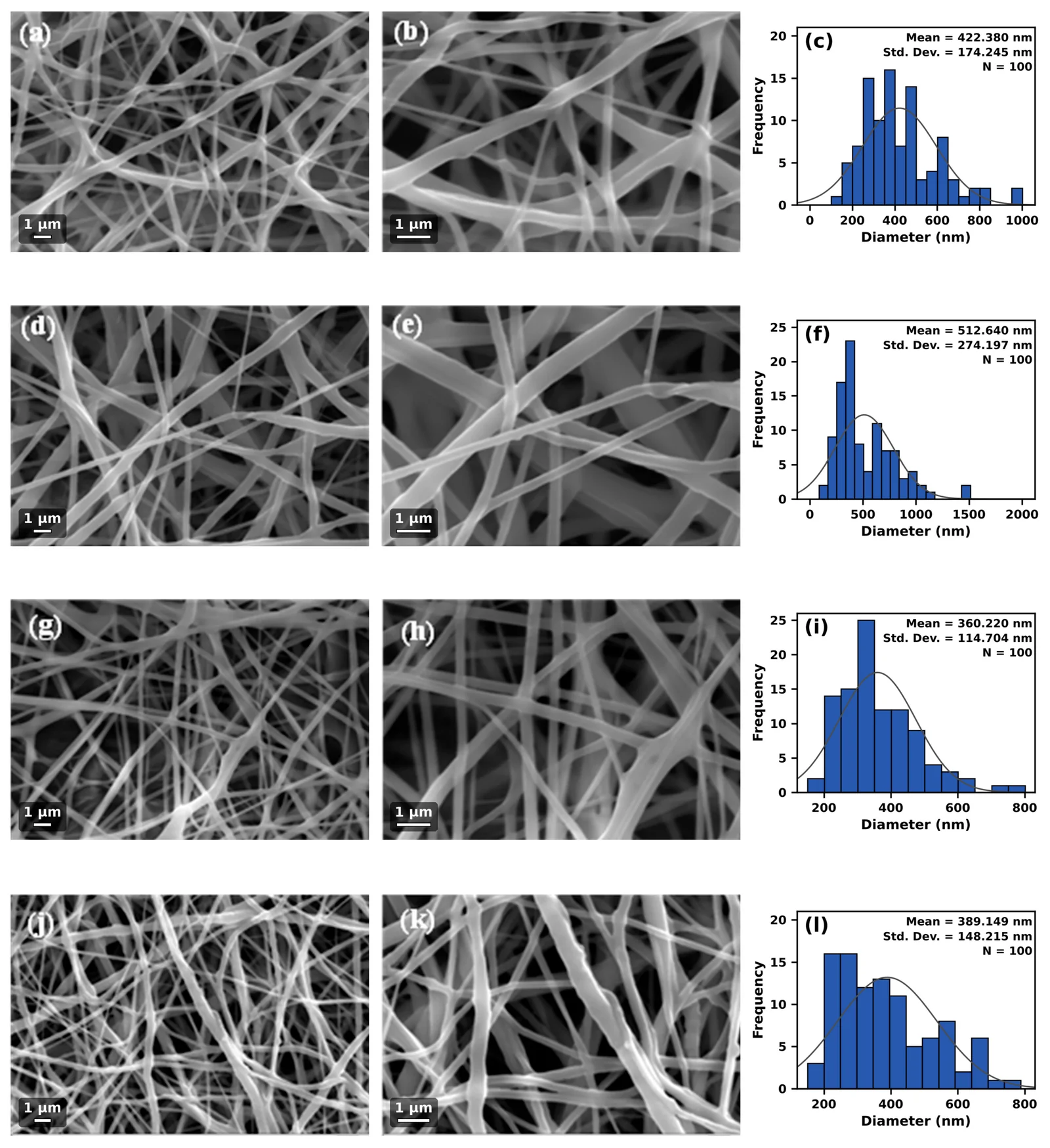
SEM images and diameter distributions of PVA-PCL, PVA/CS-PCL, PVA-PCL/TTO, and PVA/CS-PCL/TTO nanofibers: (**a**,**b**) PVA-PCL nanofibers at ×5000 and ×10,000 magnifications, respectively; (**c**) diameter distribution of PVA-PCL nanofibers; (**d**,**e**) PVA/CS-PCL nanofibers at ×5000 and ×10,000 magnifications, respectively; (**f**) diameter distribution of PVA/CS-PCL nanofibers; (**g**,**h**) PVA-PCL/TTO nanofibers at ×5000 and ×10,000 magnifications, respectively; (**i**) diameter distribution of PVA-PCL/TTO nanofibers; (**j**,**k**) PVA/CS-PCL/TTO nanofibers at ×5000 and ×10,000 magnifications, respectively; (**l**) diameter distribution of PVA/CS-PCL/TTO nanofibers.

The SEM images and corresponding histograms of fiber diameter distributions for PVA-PCL, PVA/CS-PCL, PVA-PCL/TTO, and PVA/CS-PCL/TTO nanofibers are shown in Figure 2. The surface morphology and diameter distributions of nanofibers produced by electrospinning were evaluated using SEM analysis. The SEM micrographs clearly demonstrated that all co-electrospun hybrid formulations (PVA-PCL, PVA/CS-PCL, PVA-PCL/TTO, and PVA/CS-PCL/TTO) possessed bead-free, continuous, smooth, and highly uniform fiber morphologies. A significant increase in fiber diameter was observed with the addition of CS to this basic structure, and the average diameter for PVA/CS-PCL nanofibers reached 512 ± 274 nm. When the literature is examined, it can be seen that this increase directly coincides with scientific mechanisms; when PVA and CS are mixed, strong hydrogen bonds are formed between the molecules (NH_2_ and OH groups), which increases the viscosity and rheological consistency index of the polymer solution. High viscosity restricted the electric field-induced jet elongation in the electrospinning process, preventing the thinning of the fibers and triggering the formation of a larger diameter fiber morphology. Our findings are consistent with literature data indicating that the addition of CS increases the fiber diameter by changing the rheological parameters [21].

In contrast, the addition of TTO to the system led to a significant decrease in fiber size. The average diameter decreased to 360 ± 114 nm for PVA-PCL/TTO and to 389 ± 148 nm for PVA/CS-PCL/TTO nanofibers. This change in morphology is explained by the presence of amphiphilic compounds in the structure of TTO, which exhibit properties similar to surfactants. These compounds significantly reduce the surface tension and viscosity of the polymer solution. This simultaneous reduction in surface tension and viscosity allows the electrical force applied during the electrospinning process to elongate the polymer jet much more without breaking it, which directly leads to the formation of finer and more uniform fiber structures [22].

Solvent composition and polymer concentration may also influence the morphological differences observed between the formulations. Compared with the PVA-only solution, the PVA/CS spinning solution differed in both polymer concentration and acetic acid/water composition. These differences may affect polymer-chain interactions, jet stretching, and the rate of solvent evaporation and fiber solidification, thereby contributing to changes in fiber diameter. Likewise, TTO incorporation into the PCL phase may modify the solution properties governing jet formation and stretching. The observed morphology may therefore reflect the combined influence of these formulation parameters.

In conclusion, the reductions in fiber diameter observed in TTO-doped samples are in complete agreement with the mechanisms of low surface tension and increased jet elongation in the literature. The fact that the observed PVA/CS-PCL/TTO nanofibers (389 nm) are slightly thicker than the PVA-PCL/TTO nanofibers containing only TTO (360 nm) indicates that the viscosity-enhancing effect of CS in the matrix is successfully balanced by the thinning/stretching effect of TTO.

### 3.2. FTIR Analysis of the Nanofibrous Mats

**Figure 3:**
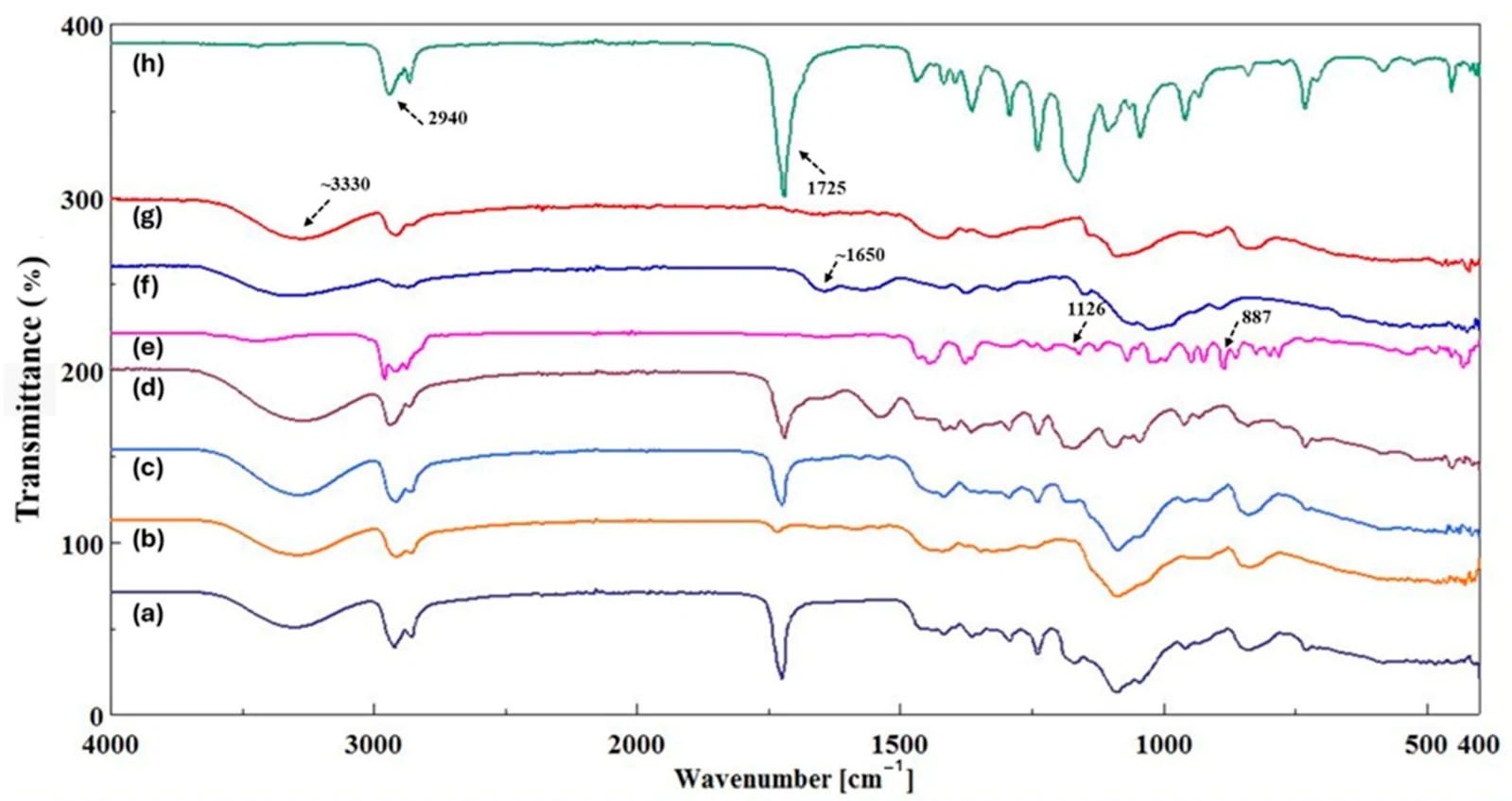
FTIR spectra of (a) PVA/CS-PCL/TTO, (b) PVA/CS-PCL, (c) PVA-PCL/TTO, (d) PVA-PCL, (e) TTO, (f) Chitosan, (g) PVA, and (h) PCL.

The FTIR spectra of the pristine components and co-electrospun mats are presented in Figure 3.

Pure PCL (h) exhibited characteristic CH_2_ stretching bands at approximately 2940 and 2865 cm^−1^, a strong ester carbonyl (C=O) stretching band near 1725 cm^−1^, and C–O–C/C–O vibrations in the 1300–1100 cm^−1^ region [23]. Pure PVA (g) showed a broad O–H stretching band within 3600–3000 cm^−1^, together with C–H stretching near 2900 cm^−1^ and C–O vibrations in the fingerprint region. Chitosan (f) displayed a broad O–H/N–H stretching band, together with amide I and amide II/amino-related absorptions at approximately 1650 and 1590 cm^−1^, respectively. In the PVA/CS-containing mats, these bands were less distinctly resolved, likely because of spectral overlap, band broadening, and hydrogen-bonding interactions between PVA and CS chains [24].

Pure TTO (e) exhibited characteristic terpene-associated absorptions, including aliphatic C–H stretching at approximately 2962 cm^−1^ and C–O stretching of terpene alcohols near 1126 cm^−1^. Bands around 887 and 864 cm^−1^ have also been associated with the terpene constituents of TTO. In the TTO-containing mats, PVA-PCL/TTO (c) and PVA/CS-PCL/TTO (a), these features became less distinct because of overlap with the dominant polymer bands. Nevertheless, the spectral variations observed in the 1200–800 cm^−1^ fingerprint region, together with their correspondence to the pure TTO spectrum, support the incorporation of TTO into the fibrous structure [14].

Compared with the PVA-PCL control (d), the PVA/CS-PCL mat (b) showed changes mainly in the broad O–H/N–H and fingerprint regions following CS incorporation, whereas the PVA-PCL/TTO mat (c) exhibited additional variations in the terpene-associated fingerprint region. The PVA/CS-PCL/TTO mat (a) retained the principal characteristic bands of PCL and PVA while also displaying spectral features consistent with the presence of CS and TTO. The absence of new and distinct absorption bands suggests that CS and TTO were predominantly incorporated through physical interactions, with no clear evidence of new covalent bond formation [25]. Consequently, these observations suggest that the components were incorporated into the hybrid nanofibrous mats without major changes to their characteristic chemical structures.

### 3.3. Thermal Analysis of the Nanofibrous Mats

**Figure 4:**
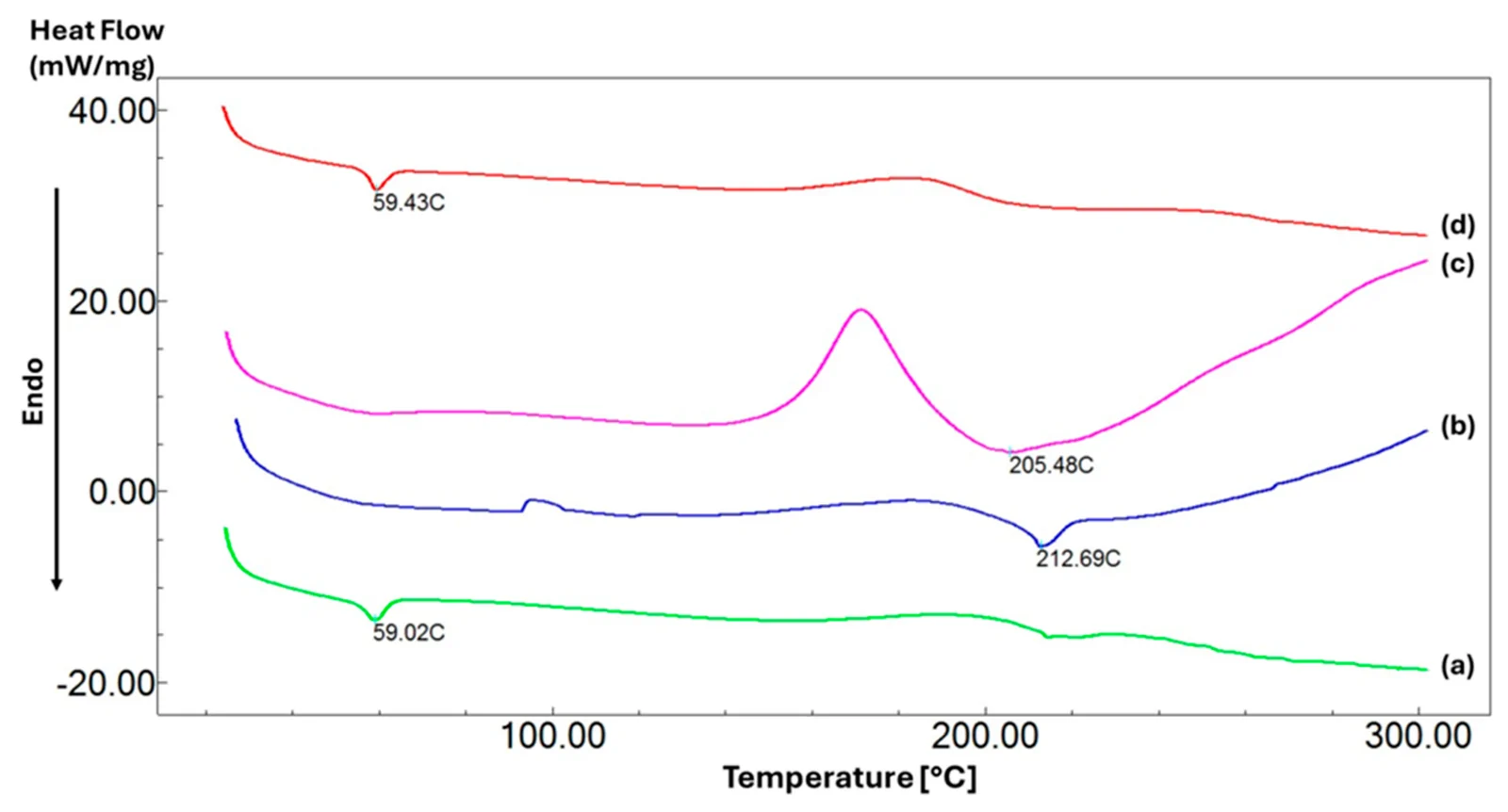
DSC thermograms of the co-electrospun nanofibrous mats: (a) PVA-PCL, (b) PVA-PCL/TTO, (c) PVA/CS-PCL, and (d) PVA/CS–PCL/TTO.

The DSC thermograms of the co-electrospun nanofibrous mats are presented in Figure 4.

The PVA-PCL and PVA/CS-PCL/TTO formulations exhibited distinct endothermic peaks at 59.02 °C and 59.43 °C, respectively. These transitions were attributed to the melting of the semicrystalline PCL domains and are consistent with the melting-temperature range commonly reported for electrospun PCL fibers [11,23]. In contrast, a clearly resolved PCL melting peak was not observed for the PVA-PCL/TTO and PVA/CS-PCL mats within this temperature range. In these two formulations, the PCL melting response may have been too weak or broad to be clearly resolved in the thermograms. However, the absence of a well-resolved peak should not be interpreted as evidence of the complete loss of the PCL crystalline phase.

Further composition-dependent differences were observed in the higher-temperature region. The PVA-PCL/TTO mat displayed an endothermic event at 212.69 °C, which was primarily associated with the PVA-rich fiber population and falls within the thermal-transition range reported for electrospun PVA-based materials [26]. The PVA/CS-PCL mat exhibited a more complex profile, comprising a broad upward-directed event at approximately 165–175 °C followed by an endothermic minimum at 205.48 °C. Considering the downward endothermic convention used in Figure 4, the first event represents an exothermic response and may be related to thermally induced chain reorganization. The subsequent endothermic event likely reflects overlapping thermal processes within the PVA/CS-rich fiber population. Because dehydration, chain reorganization, melting, and the onset of CS-associated thermal changes may occur within partially overlapping temperature ranges, these events cannot be unequivocally assigned to a single process based on DSC alone [27,28].

The PVA/CS-PCL/TTO mat exhibited a broader and less-resolved response in the higher-temperature region, without a distinct endothermic minimum comparable to those observed at 205.48 °C and 212.69 °C. This broader response suggests composition-dependent changes in the overall thermal behavior of the hybrid nanofibrous mats. Such changes may arise from physical interactions among PVA, CS, and TTO constituents that affect polymer-chain mobility and packing. Nevertheless, the possible volatilization or thermal loss of TTO components during heating should also be considered when interpreting this region [12].

The occurrence of PCL-associated and PVA/CS-associated thermal responses is compatible with the simultaneous dual-nozzle co-electrospinning configuration employed in this study. The hydrophilic PVA or PVA/CS solution and the hydrophobic PCL or PCL/TTO solution were processed through separate nozzles, and the independently generated fibers were brought together only upon deposition onto the collector. Consequently, the thermograms are consistent with the presence of compositionally distinct but physically integrated fiber populations rather than a completely homogeneous molecular blend [4,5]. However, DSC alone cannot confirm the spatial distribution or degree of phase separation within the mats; therefore, this interpretation should be considered together with the morphological and spectroscopic findings.

Overall, the DSC results demonstrate that the thermal behavior of the co-electrospun mats varied considerably with their composition. The characteristic PCL melting region remained detectable in the PVA-PCL and PVA/CS-PCL/TTO formulations, whereas the higher-temperature responses associated with the PVA- and PVA/CS-rich fiber populations were modified by the incorporation of CS and TTO. These findings suggest composition-dependent differences in thermal behavior that may be associated with changes in polymer-chain organization and intermolecular interactions within the nanofibrous mats. The thermal transition temperatures and phase characteristics derived from the DSC analysis for all hybrid nanofibrous formulations are summarized in Table 2.

### 3.4. Tensile Tests of the Nanofibrous Mats

**Figure 5:**
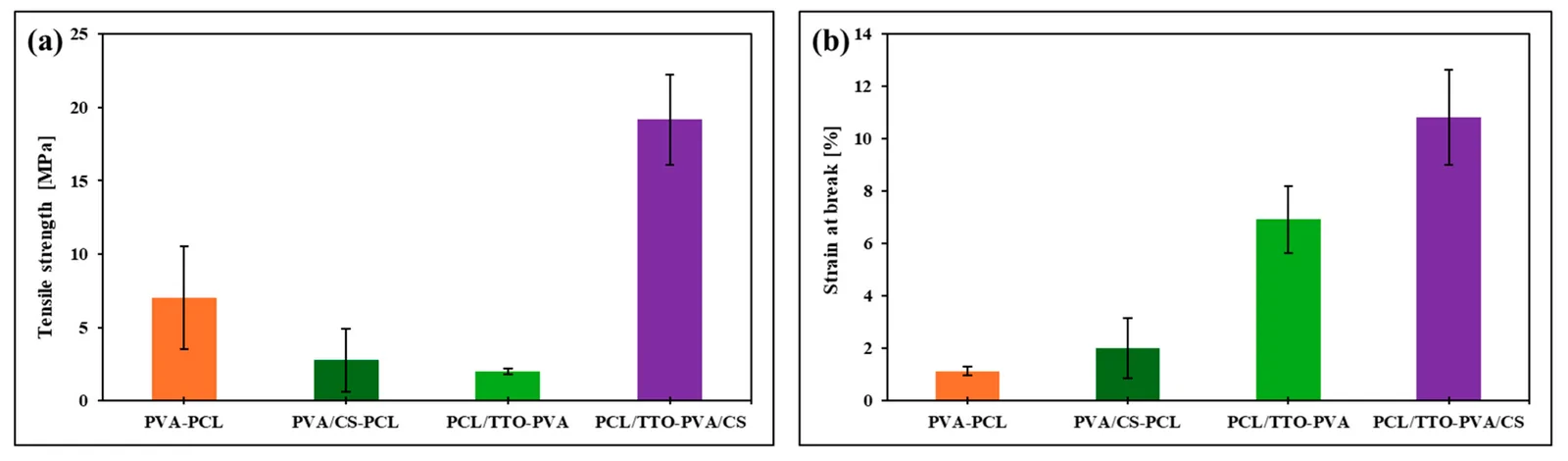
Tensile properties of the co-electrospun hybrid nanofibrous mats: (**a**) tensile strength and (**b**) strain at break. All experiments were performed in triplicate, and the results are expressed as mean ± standard deviation (SD) (*n* = 3).

The tensile properties of the co-electrospun hybrid nanofibrous mats were markedly influenced by their composition (Figure 5).

The PVA-PCL mat exhibited a tensile strength of 7.03 ± 3.49 MPa and a strain at break of 1.13 ± 0.17%, indicating a comparatively high load-bearing capacity but limited extensibility. Following CS incorporation, the tensile strength decreased to 2.76 ± 2.17 MPa, whereas the strain at break increased to 2.01 ± 1.16%. This response suggests that CS altered the internal organization of the hybrid nanofibrous mat, reducing its resistance to maximum tensile loading while providing a modest improvement in deformability. The broader fiber-diameter distribution observed by SEM for PVA/CS-PCL (512 ± 274 nm) may have generated local structural heterogeneities and less efficient stress transfer throughout the fibrous network. Moreover, the CS-related changes detected by FTIR and DSC indicate intermolecular interactions and altered organization within the PVA/CS-rich fiber population, which may have further influenced its mechanical response. Similar composition-dependent variations in strength and flexibility have been reported for electrospun PVA/CS mat [28]. More generally, the tensile behavior of electrospun nanofibrous mats is governed not only by polymer composition but also by fiber morphology, diameter distribution, alignment, porosity, and interfiber junctions [23].

TTO incorporation produced a pronounced strength–extensibility trade-off. The PVA-PCL/TTO mat exhibited the lowest tensile strength (2.00 ± 0.17 MPa) but a substantially higher strain at break (6.93 ± 1.28%) than the PVA-PCL mat. This finding indicates that TTO increased the deformability of the nanofibrous mat while decreasing its resistance to maximum tensile loading. Since FTIR analysis showed no evidence of new covalent bond formation, TTO was most likely retained through physical interactions within the PCL-rich fibers. The low-molecular-weight terpene constituents of TTO may therefore have acted as plasticizing or interfacial modifiers, increasing polymer-chain mobility while reducing cohesive interactions and the efficiency of stress transfer. This interpretation is consistent with the TTO-induced decrease in apparent viscosity observed in the rheological analysis, the formation of finer fibers detected by SEM (360 ± 114 nm), and the changes in polymer-chain organization indicated by DSC. Previous studies have likewise demonstrated that TTO incorporation can modify the mechanical behavior of PVA/CS- and PCL-containing polymeric systems [12,25].

Notably, the PVA/CS-PCL/TTO mat exhibited the highest tensile strength (19.17 ± 3.06 MPa) and strain at break (10.82 ± 1.81%) among all investigated formulations. This marked mechanical enhancement can be attributed to synergistic physical interactions and robust intermolecular hydrogen bonding between the amine and hydroxyl groups of chitosan and PVA, coupled with the uniform co-deposition of hydrophobic PCL/TTO nanofibers. Furthermore, the dual-nozzle co-electrospinning process facilitated dense fiber–fiber interlocking and balanced load distribution throughout the hybrid network, thereby significantly enhancing resistance to uniaxial deformation [29]. Unlike the individual incorporation of CS or TTO, their combined presence resulted in a nanofibrous mat capable of sustaining a higher tensile load while simultaneously undergoing greater deformation before failure. This enhanced mechanical response may reflect the collective effects of fiber morphology, interfiber interactions, and the distribution of the compositionally distinct fiber populations within the co-deposited mat. Intermolecular interactions within the PVA/CS-rich fibers may have contributed to network cohesion, whereas physically incorporated TTO within the PCL-rich fibers may have increased chain mobility and reduced brittleness. The simultaneous deposition of these distinct fiber populations may therefore have facilitated stress redistribution throughout the hybrid fibrous network [4]. This plasticizing action of TTO is primarily governed by its low-molecular-weight terpene constituents, which physically insert between polymer chains, weaken intermolecular cohesion, and increase the free volume of the polymer matrix. This mechanism is consistent with findings by Zhang et al., who reported that tea tree essential oil acts as an effective natural plasticizer in electrospun hydrophobic matrices, enhancing chain mobility and significantly improving the extensibility of the fibrous network [30]. Overall, the combined formulation provided the most favorable balance between tensile strength and extensibility under the applied dry-state testing conditions. This mechanical behavior complements its controlled swelling and mass-loss profiles, enhanced wettability, and favorable HDF-cell response. The tensile results were obtained under dry conditions. Future wet-state testing could provide further insight into whether the observed strength improvement is retained after exposure to wound fluid.

### 3.5. Swelling and Degradation Tests of the Nanofibrous Mats

**Figure 6:**
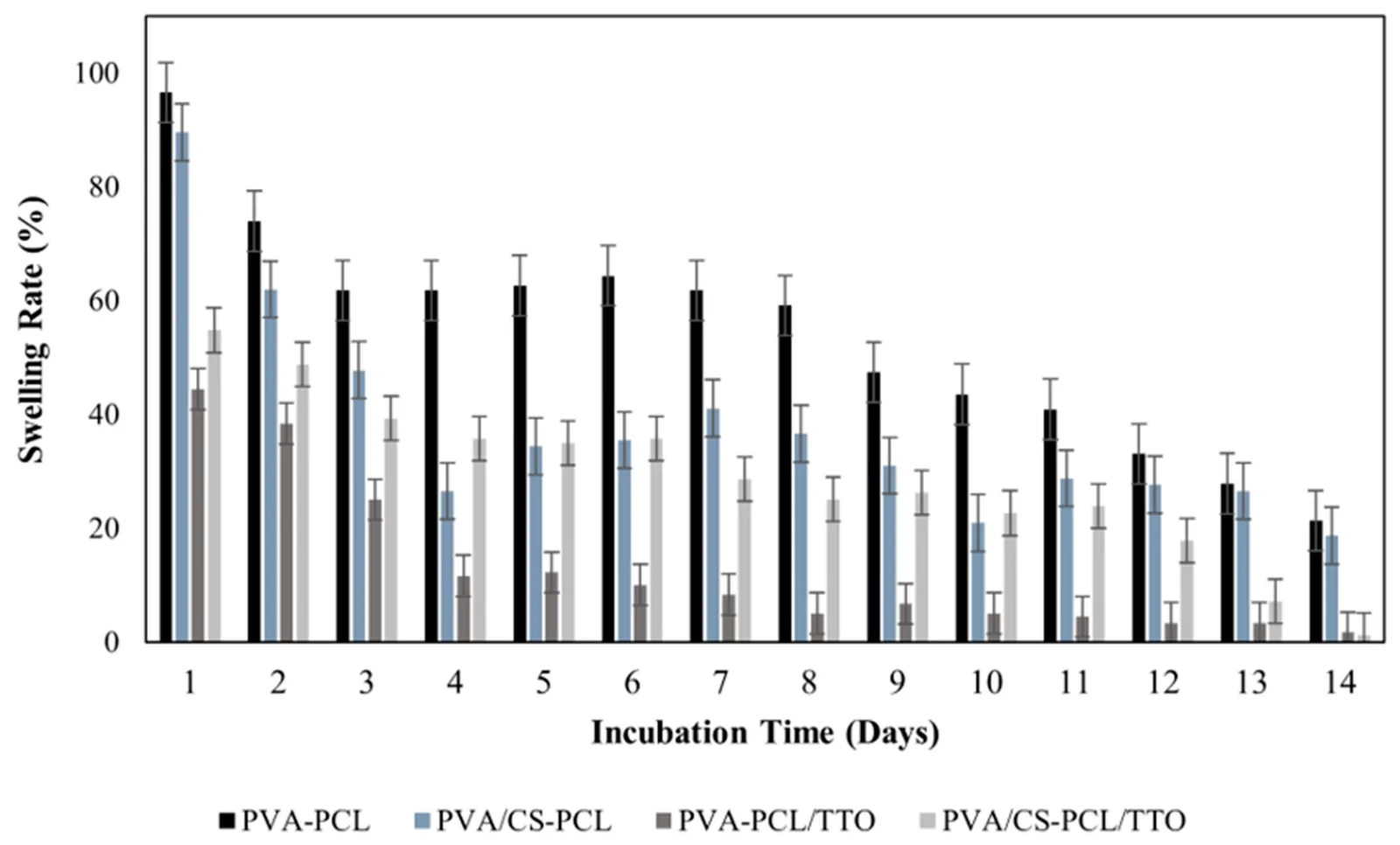
Time-dependent swelling kinetics of nanofibrous mats evaluated at regular 24-h intervals over a 14-day incubation period in PBS.

**Figure 7:**
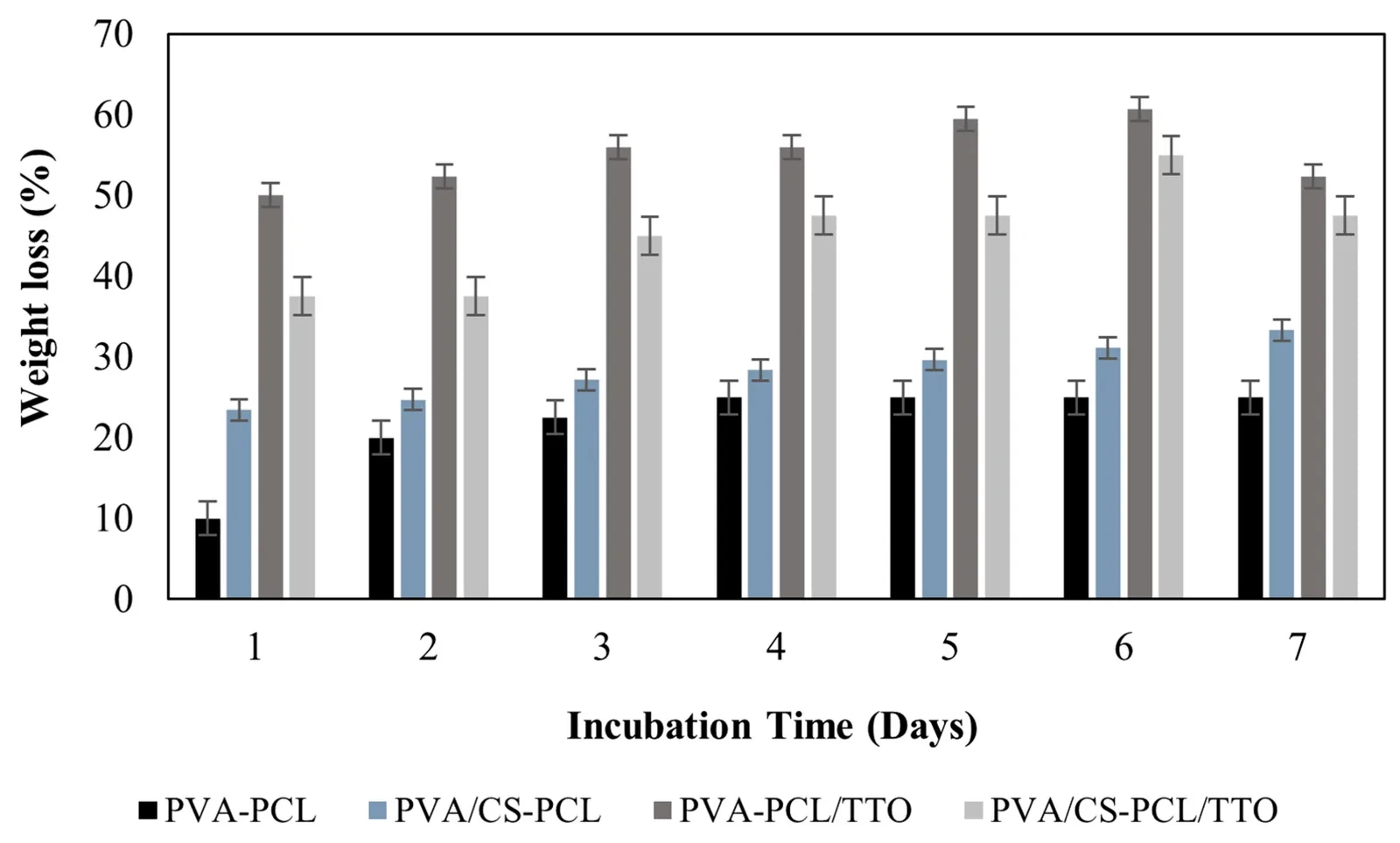
In vitro weight loss profiles of nanofibrous mats evaluated over a 7-day incubation period in PBS.

The swelling profiles of the prepared composite mats during the 14-day incubation period are presented in Figure 6, while their degradation behaviors over a 7-day period are shown in Figure 7.

On the first day of incubation, the highest swelling ratio was measured in the neat PVA-PCL mat at approximately 96.5%, while the addition of chitosan to the structure reduced this value to approximately 90% (PVA/CS-PCL). The reduction in swelling ratio in the control groups compared to the pure mats due to the presence of chitosan is explained by hydrogen bond interactions and polymeric network density within the matrix. Chitosan forms strong intermolecular hydrogen bonds with PVA chains in the amorphous phase through its primary amino and hydroxyl groups. DSC analysis in the literature demonstrates that chitosan addition reduces the crystallization degree of pure PVA fibers from 81.02% to 28.99%. While expansion of amorphous regions normally facilitates water infiltration, these multifaceted physical interactions between chitosan and PVA increase the physical cross-linking density of the matrix, tightening the polymeric network structure, and the water-insoluble nature of chitosan limits its water-holding capacity. This tight network structure physically restricts the free movement of water molecules within the matrix and prevents excessive stretching of the polymer chains due to water absorption; thus, the water-holding capacity of the control mat containing chitosan remains at a more controlled level [27].

The fact that the inclusion of hydrophobic TTO in the matrix dramatically reduces water uptake capacities from approximately 90–96% to 45–55% is directly related to the highly hydrophobic chemical nature of the oil [31]. TTO consists of monoterpene components such as terpinen-4-ol (35–48%), γ-terpinene (15–28%), and α-terpinene (6–12%), which are water-repellent, have high log *p* values (log *p* ≈ 2.5–5.3), and are practically insoluble in water [32]. These non-polar active components prevent water molecules from interacting with hydrophilic groups within the polymer matrix. The oil droplets occupy physical volume in the mat matrix, blocking gaps through which water molecules could penetrate and preventing the hydration of the polymer chains. Because water cannot penetrate the matrix, the mobility of the polymer chains is restricted, and the mat’s tendency to swell when exposed to water is physically suppressed [31]. When comparing the groups containing TTO, the chitosan-containing mat (PVA/CS-PCL/TTO) exhibited a higher water uptake capacity (~55% vs. ~45%) on day 1 compared to the chitosan-free mat (PVA-PCL/TTO). This is due to chitosan’s role as an emulsion stabilizer and oil trap. In the absence of chitosan (PVA-PCL/TTO), hydrophobic TTO domains localized within the PCL fiber population physically occupy matrix free volume and increase local hydrophobic interactions. Rather than forming a completely impermeable surface layer, these hydrophobic regions restrict bulk fluid penetration into the interior matrix, thereby controlling overall swelling. However, when chitosan is incorporated into the structure (PVA/CS-PCL/TTO), it indirectly modulates matrix hydration by maintaining the structural stability and water affinity of the hydrophilic PVA/CS fiber network. Consequently, the water uptake capacity moderately increases to ~55% (compared to ~45% in PVA-PCL/TTO), establishing a balanced and stable swelling profile. In the twin-nozzle co-electrospinning setup, chitosan is localized within the hydrophilic PVA fibers, while TTO is embedded within the hydrophobic PCL fibers. Rather than directly encapsulating or forming hydrogen bonds with TTO droplets, the hydrophilic functional groups of chitosan within the PVA matrix enhance the overall structural and hydrophilic network stability [31,32].

This co-presence of hydrophilic CS-containing fibers adjacent to hydrophobic PCL-TTO fibers prevents the collapse of the porous network and ensures controlled water infiltration without allowing the hydrophobic TTO phase to completely block fluid uptake, thereby enabling a controlled, stable, and balanced swelling profile. The partial affinity of the hydrophilic functional groups in chitosan’s own structure for water molecules is maintained; this prevents the TTO-containing mat from completely closing off to water, enabling a controlled, stable, and balanced swelling profile [31]. The continuous and gradual decrease in the water uptake capacities of all groups as the incubation period progresses from day 1 to day 14 is explained by the degradation, mass loss, and solubility mechanisms of the polymer matrix in the aqueous environment. Hydrophilic PVA components, which have very high water solubility, gradually dissolve and move away from the matrix surface and amorphous regions when they remain in contact with water for a long time. As the mats are kept in water, they undergo mass loss; this causes the polymeric network structure to weaken over time and the hydrophilic polymer fractions, which represent the water uptake capacity, to decrease [27]. As the dissolved PVA separates from the matrix and less soluble fractions remain, the mats’ abilities to attract water molecules and swell gradually decrease, and the graph regresses to minimum levels at the end of day 14.

The findings show that the addition of CS and volatile TTO to the polymeric matrix has statistically significant effects on the weight loss rate and total mass loss. When the control groups without TTO were examined, it was determined that the inclusion of chitosan in the matrix (PVA/CS-PCL) significantly increased the weight loss rate. Indeed, the mass loss, which was 10% in the chitosan-free PVA-PCL mat on the 1st day of incubation, increased to 23% in the presence of chitosan, and from 25% to 33% at the end of the 7th day. This role of chitosan in increasing mass loss is explained by the amorphous transformations in the crystalline phase of the polymeric matrix. Pure PVA, thanks to its regular semi-crystalline structure, exhibits a high degree of crystallization of 81.02% in the literature. However, the primary amino (-NH_2_) and hydroxyl (-OH) functional groups of chitosan added to the formulation prevent the regular packing of the PVA chains by forming strong hydrogen bonds with the PVA chains in the amorphous phase, reducing the degree of crystallization to 28.99% [27,31]. The large amorphous regions replacing the crystalline phase facilitate the penetration of water molecules into the matrix and polymer chain mobility. As a result, the hydrophilic PVA components in the amorphous regions in contact with water dissolve rapidly, separating from the matrix and resulting in a higher mass loss in the early stages. On the other hand, the inclusion of hydrophobic and volatile TTO in the structure dramatically increased the weight loss rate. The chitosan-free PVA-PCL/TTO mat showed the fastest weight loss tendency, exhibiting approximately 50% mass loss on the first day of incubation and reaching the maximum loss level (~61%) on the 6th day. The significant increase in mass loss due to the addition of TTO stems from both the volatile chemical nature of the oil and the morphological changes it induces in the matrix. TTO consists primarily of volatile monoterpenes with low molecular weight and high vapor pressure (~2.1 kPa), such as terpinen-4-ol (35–48%), γ-terpinene (15–28%), and α-terpinene (6–12%). The sudden mass loss spike observed in the first 24 h is directly related to the rapid evaporation of these volatile components from the matrix surface in an open environment and their burst release [32]. Simultaneously, the thermodynamic incompatibility between the hydrophilic polymer phase and the hydrophobic TTO leads to phase separation during the mat drying process. The coalescence and migration of emulsified oil droplets to the surface and the evaporation of the solvent leave dense microvoids and structural irregularities in the cross-section. This resulting porous structure accelerates water diffusion, facilitating the wash-out process of hydrophilic polymer fractions [31]. Remarkably, the inclusion of chitosan in the TTO-containing system (PVA/CS-PCL/TTO) significantly limited this excessive loss. In this group, the mass loss on day 1 remained at 37% and the loss at the end of day 7 remained at 47%, resulting in a much more balanced and stable profile compared to its chitosan-free counterpart. The stabilizing effect of chitosan on limiting mass loss is explained by its ability to form a highly stable, water-insoluble physical network with PVA (as shown by restricted crystallization), which prevents the rapid dissolution and wash-out of the hydrophilic phase, thereby maintaining the overall structural integrity of the co-deposited mat. The stabilizing effect of chitosan on limiting mass loss is explained by its ability to form a highly stable, water-insoluble physical network with PVA, which prevents the rapid disintegration and wash-out of the hydrophilic fiber phase, maintaining the 3D integrity of the co-deposited mat. By preserving the physical structure of the hybrid mat, the CS-PVA network indirectly restricts the fast erosion-driven loss of TTO from the neighboring PCL fibers. This structural support prevents the collapse of the porous channels created by oil volatilization and phase separation, leading to a more controlled and stable overall mass loss over the 7-day incubation period.

It should be noted that the observed mass loss in PBS encompasses both structural polymer erosion and the partial dissolution of uncrosslinked hydrophilic components (PVA and chitosan), alongside the release of incorporated additives (TTO and Tween 80). The slight stabilization in mass loss observed between days 6 and 7 indicates that the readily soluble fractions reached dissolution equilibrium; minor fluctuations at these late timepoints can also be attributed to PBS buffer salt deposition onto the porous nanofibrous scaffold during drying. Furthermore, because conventional swelling calculations rely on the initial dry weight (W0), ongoing mass loss leads to a slight underestimation of the intrinsic water-retention capacity of the remaining hydrated matrix.

### 3.6. Release Tests of the Nanofibrous Mats

**Figure 8:**
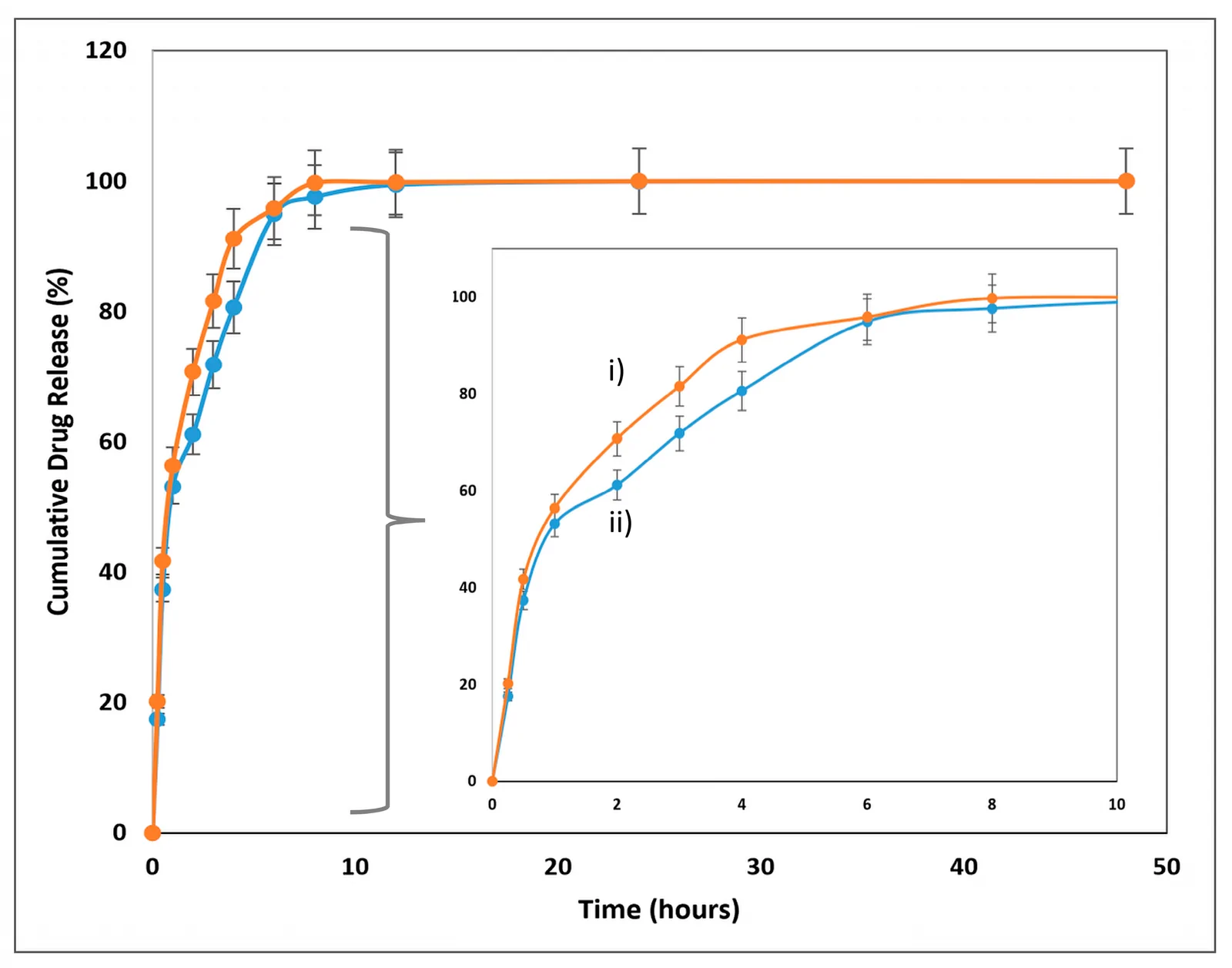
In vitro drug release behavior of TTO from PVA/CS-PCL/TTO (i) and PVA-PCL/TTO nanofibers (ii). All experiments were performed in triplicate, and the results are expressed as mean ± standard deviation (SD) (*n* = 3).

The in vitro drug release results obtained to evaluate the release behavior of TTO in PVA-PCL/TTO and PVA/CS-PCL/TTO nanofibers are presented in Figure 8.

The in vitro drug release results obtained to evaluate the release behavior of TTO in PVA-PCL/TTO and PVA/CS-PCL/TTO nanofibers are presented in Figure 8. After the calibration curve for TTO was plotted at a wavelength of 265 nm with an R^2^ value of 0.9977, the drug release results were evaluated (Figure S1). The release profiles of TTO-loaded nanofibers exhibited different behavior in both formulations as shown in Figure 8. During the first 30 min, 37.38% and 41.75% of the loaded TTO were released from the PVA-PCL/TTO and PVA/CS-PCL/TTO nanofibers, respectively. Following this rapid initial phase, the release rate slowed significantly. TTO release was practically complete in both groups by the 8th hour (97.63% and 99.75%) and this level of release was maintained up to 48 h. This rapid release profile is consistent with the structural properties of the nanofibers. This is because the addition of TTO reduced the fiber diameter to 360 ± 114 nm and 389 ± 148 nm, thereby increasing the surface-to-volume ratio, and significantly improved wettability by lowering the water contact angle from 74.78° ± 3.16° to 46.69° ± 2.80° (for the CS-free formulations) and from 74.85° ± 3.69° to 57.05° ± 1.40° (for the CS-containing formulations). Furthermore, the rapid dissolution of the hydrophilic PVA phase in the PBS medium creates diffusion channels within the matrix at an early stage, as evidenced by the 50% and 37% mass losses observed during the first 24 h.

Chitosan-containing nanofibers exhibited higher cumulative release at all points examined. While the difference in the release behavior of TTO was limited to the burst phase (37.38% vs. 41.75%), it became more pronounced in the diffusion phase, reaching its highest level at the 4-h mark (80.66% vs. 91.18%). This is explained by the fact that in the twin-nozzle co-electrospinning system, TTO is present in the PCL phase, while chitosan is present in the PVA phase. Since the two components are present in different fiber populations, the oil stabilization mechanism based on chitosan-terpinen-4-ol hydrogen bond interactions reported by Jiang et al. in a chitosan/PVA Pickering emulsion system does not apply in this system [15]. Instead, the effect of chitosan manifests indirectly through the hydrophilic phase. The hydrogen bonds formed between chitosan and PVA chains expand the amorphous regions, facilitating water penetration [27]. Indeed, the fact that the chitosan-containing formulation exhibits a higher water uptake capacity (~55% vs. ~45%) supports this interpretation. In contrast, the phase separation that occurs during drying in chitosan-free nanofibers leads to the migration of oil to the surface, forming a hydrophobic layer that restricts water passage. The fact that this formulation exhibits slower release despite having a finer fiber diameter and a lower contact angle indicates that the rate-determining step in the system is not surface wettability, but rather the hydration and erosion behavior of the hydrophilic phase.

The profiles obtained are consistent with the behavior reported in the literature for PVA-based electrospun carriers. It has been reported that the release of tetracycline hydrochloride from PVA/chitosan nanofibers occurs in a burst-like manner during the first 2 h, then slows down to reach a saturation level corresponding to approximately 80% of the loaded amount, and that this level is maintained for 24 h [33]. While in most of these systems, the release remaining at a plateau level indicates the presence of a fraction trapped within the matrix, in the present study, the cumulative release reaching 100% in both formulations indicates that the releasable TTO fraction was fully released within the test period. On the other hand, Santos et al. reported only a 6.8% release within the first 24 h in a system where TTO was loaded onto hydrophobic PHBV nanofibers; the difference stems from the structural distinction between non-swelling hydrophobic carriers and a hybrid architecture containing a hydrophilic PVA/CS phase [13]. In terms of wound dressing applications, the high-concentration release provided in the first few hours is advantageous in that it ensures rapid and effective antimicrobial protection during the early wound phase, when the risk of microbial colonization is highest [3,12].

**Figure 9:**
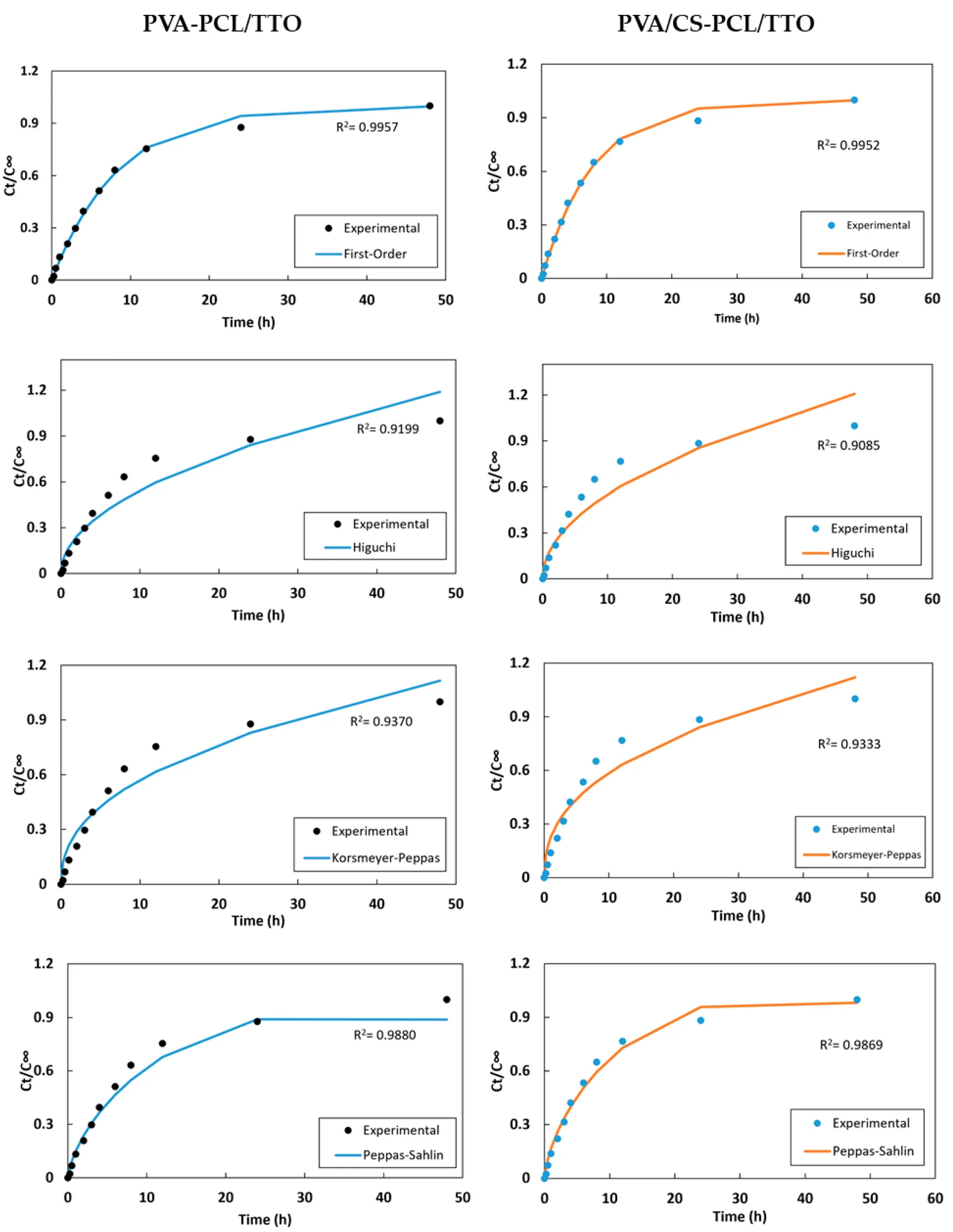
In vitro drug release kinetic behavior of TTO from PVA-PCL/TTO and PVA/CS-PCL/TTO nanofibers.

To determine the release mechanism of TTO, the release data were fitted to first-order, Higuchi, Korsmeyer–Peppas, and Peppas–Sahlin models (Figure 9 and Table 3). The best fit was obtained with first-order kinetics in both formulations (R^2^ = 0.9957 for PVA-PCL/TTO and R^2^ = 0.9952 for PVA/CS-PCL/TTO). This result indicates that the release rate is proportional to the amount of TTO remaining in the fiber. The fact that the Peppas–Sahlin model also showed a high degree of fit (R^2^ = 0.9880 and 0.9869) indicates that the release occurs not only through diffusion but also through the swelling and erosion of the hydrophilic phase. Similarly, it has been reported that the release of volatile components from PVA-based electrospun fibers is best described by the Peppas–Sahlin model [34]. In chitosan/PEO nanofibers, a diffusion–wear-out mechanism has been identified in which the rapid dissolution of the water-soluble phase drives early release, while the swelling and partial wear-out of chitosan drive subsequent release [35]. The lower fit of the Higuchi (R^2^ = 0.9199 and 0.9085) and Korsmeyer–Peppas (R^2^ = 0.9370 and 0.9333) models supports the conclusion that the release cannot be explained by pure Fickian diffusion. This finding is consistent with the anomalous (non-Fickian) release behavior reported for PCL/chitosan nanofibers [36]. The fact that the R^2^ values for the two formulations are very close indicates that chitosan does not alter the release mechanism but merely increases the release rate by enhancing water uptake.

### 3.7. Water Contact Angles of the Nanofibrous Mats

The surface wettability of the electrospun nanofibrous formulations was evaluated by water contact angle measurements. Table 4 summarizes the corresponding contact angle values along with droplet images for PVA-PCL, PVA-PCL/TTO, PVA/CS-PCL, and PVA/CS-PCL/TTO.

The surface wettability of the electrospun nanofibrous formulations was evaluated by water contact angle measurements. Table 4 summarizes the corresponding contact angle values along with droplet images for PVA-PCL, PVA-PCL/TTO, PVA/CS-PCL, and PVA/CS-PCL/TTO. Surface wettability and water contact angle (WCA) properties of electrospun nanofiber mats are critical physical parameters that directly affect cell adhesion, proliferation, and consequently tissue regeneration [37]. Experimental measurements revealed that the contact angle of pure PVA-PCL electrospun fibers was 74.78° ± 3.16°, indicating that the material possesses a hydrophilic/wettable surface characteristic. In the PVA/CS-PCL formulation obtained by adding chitosan, a cationic polyelectrolyte, to the matrix, it was determined that the contact angle reached a value of 74.85° ± 3.64°, representing a statistically insignificant difference (*p* > 0.05). This indicates that the inclusion of chitosan did not alter the baseline wettability of the hybrid mat. Similar polymer blending studies in the literature support this stable and mildly limiting effect of chitosan addition on surface wettability. For example, in the study conducted by Czibulya et al., it was shown that while the contact angle of pure PVA nanofibers that dissolve quickly in water was 25.4° ± 2.6°, the addition of chitosan to the structure and cross-linking with citric acid increased the contact angle in a controlled manner, reaching a value of 51.8° ± 4.3° in the highest chitosan group (J2). This is attributed to chitosan’s less hydrophilic nature compared to PVA and the cross-links formed within the polymeric network [38]. Similarly, Al-Madhagy et al. reported an initial contact angle of 72.3° ± 1.9° for the chitosan/PVA nanofiber mats (70/30 ratio) they prepared. This finding is highly consistent with the contact angle values (~74.5°) obtained for the PVA/CS-PCL formulation. In the same study, it was stated that the contact angle decreased to 29.5° ± 0.7° within 60 s thanks to the rapid interactions of the free amino and hydroxyl groups in the chitosan and PVA chains with the water droplet, and that the mat had a high wettability dynamic [37]. One of the most striking findings of our study is the significant reduction in contact angle resulting from the integration of TTO into electrospun formulations. Adding TTO to the PVA-PCL matrix reduced the water contact angle to 46.69° ± 2.80°, resulting in a highly hydrophilic and highly wettable surface. A similar synergistic effect was observed in the chitosan-containing PVA/CS-PCL/TTO formulation, resulting in a reduced contact angle of 57.05° ± 1.40°. This wettability-enhancing effect of TTO is in excellent agreement with characterization studies of essential oil-added electrospun systems in the literature. Santos et al. reported that when they integrated TTO into a PHBV nanofiber mat, the water contact angle decreased by 54%, from 90.2° ± 6.2° (hydrophobic character) to 48.6° ± 2.4° (hydrophilic character). Researchers attributed this decrease to the spreading of lipophilic terpene constituents across the fiber surface, reducing surface tension [13]. In parallel, a study conducted by Almeida et al. showed that the contact angle of Ecovio electrospun fibers, which initially had a hydrophobic character (120° ± 2°), was reduced to 69° ± 1° with the addition of TTO, and the material acquired a hydrophilic structure. This important hydrophilic transition has been attributed to the presence of terpinen-4-ol alcohol, the main active component of tea tree oil. This alcohol component increases the polarity of the fiber surface and facilitates the formation of strong intermolecular hydrogen bonds with water molecules [22]. In addition to chemical composition, this hydrophilicity transition is closely linked to the fiber morphology. As demonstrated in the SEM analysis, TTO incorporation significantly reduced the fiber diameters, thereby increasing the surface-area-to-volume ratio and modulating the surface roughness of the co-deposited mat, which further facilitates capillary water capillary-driven spreading across the fibrous network.

### 3.8. Rheological Characterizations of the Solutions

**Figure 10:**
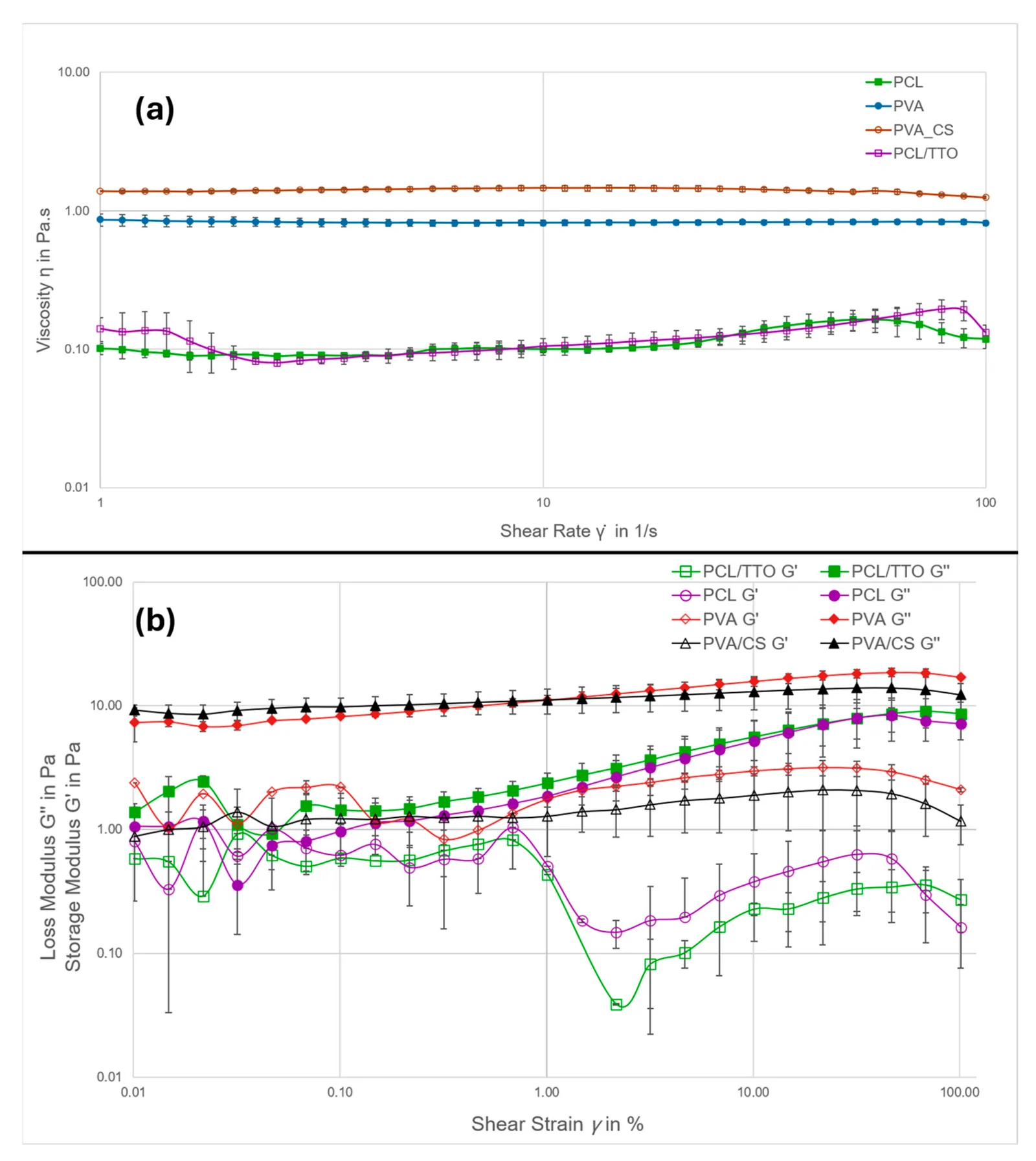
Rheological behavior of the electrospinning formulations: (**a**) apparent viscosity as a function of shear rate; (**b**) amplitude sweep curves (G′ and G″) of PVA, PVA/CS, PCL, and PCL/TTO.

The rheological characteristics of the electrospinning formulations are presented in Figure 10. The apparent viscosity profiles as a function of shear rate are shown in Figure 10a, providing insight into the flow behavior of the precursor solutions. In addition, the viscoelastic response of the formulations was assessed by amplitude sweep measurements, with the G′ and G″ curves shown in Figure 10b.

The rheological stability and flow behavior of polymeric solutions used in the electrospinning process play a decisive role in the morphology of fibers obtained through stable jet formation [39]. The viscosity profiles of the formulations were evaluated over a shear-rate range of 1–100 s^−1^. PVA/CS exhibited the highest viscosity behavior, followed by PVA, whereas PCL and PCL/TTO showed considerably lower viscosity values. At a shear rate of 1 s^−1^, the viscosities of PCL, PCL/TTO, PVA, and PVA/CS were 0.10 ± 0.01, 0.14 ± 0.03, 0.86 ± 0.09, and 1.39 ± 0.02 Pa·s, respectively. At 100 s^−1^, the corresponding values were 0.12 ± 0.02, 0.13 ± 0.02, 0.82 ± 0.03, and 1.25 ± 0.02 Pa·s. The viscosities of PVA and PVA/CS remained relatively constant over the investigated shear-rate range, indicating near-Newtonian flow behavior. The viscosity of PVA decreased by only approximately 5% between 1 and 100 s^−1^, while that of PVA/CS decreased by approximately 10%. PCL and PCL/TTO also did not exhibit a consistent shear-thinning response, suggesting that their flow behavior was relatively weakly dependent on shear rate under the investigated conditions. Overall, the results indicate that the formulations were predominantly near-Newtonian rather than strongly shear-thinning within the investigated shear-rate range.

The storage (G′) and loss (G″) moduli responses obtained in dynamic amplitude sweep analyses reveal the viscoelastic character and microstructural stability of the formulations. In dilute and semi-dilute polymer emulsions reported in the literature, a viscous-liquid-like character (G″ > G′) is commonly observed, indicating that viscous properties are dominant over elastic properties [28,40]. In contrast to the viscosity results, the strain sweep provides information on the relative elastic and viscous contributions of the formulations. G″ remained higher than G′ throughout the investigated strain range for all four formulations, indicating that the formulations exhibited a predominantly viscous rather than elastic viscoelastic response. Among the tested formulations, PVA and PVA/CS exhibited higher G′ and G″ values than PCL and PCL/TTO formulations over most of the strain range. For example, at approximately 10% strain, PVA showed G′ and G″ values of 2.94 ± 0.61 and 15.75 ± 1.51 Pa, respectively. PVA/CS exhibited lower values of 1.89 ± 0.90 Pa for G′ and 12.98 ± 3.77 Pa for G″ at the same strain. In comparison, substantially lower moduli were obtained for the PCL-based formulations. At approximately 10% strain, PCL exhibited G′ and G″ values of 0.38 ± 0.25 and 5.15 ± 2.36 Pa, respectively, whereas the corresponding values for PCL/TTO were 0.23 ± 0.08 and 5.57 ± 1.98 Pa. The incorporation of TTO therefore produced relatively small changes in the viscoelastic response of the PCL-based formulation. PCL/TTO generally exhibited lower G′ values at low-to-intermediate strain levels, whereas G″ values were slightly higher over much of the investigated strain range. Although these differences suggest a slight tendency toward a lower elastic contribution and a relatively higher viscous contribution following TTO incorporation, the variability among replicates indicates that this effect should be interpreted cautiously.

Overall, the rheological results demonstrate clear differences among the PVA- and PCL-based solutions in both their flow and viscoelastic behavior. PVA and PVA/CS exhibited substantially higher viscosity and viscoelastic moduli than the PCL-based formulations, while all formulations showed a predominantly viscous response based on the consistently higher G″ values. The incorporation of TTO into PCL produced relatively modest changes in both viscosity and viscoelastic properties rather than a uniform alteration of the rheological behavior.

### 3.9. In Vitro Assessment of Cell Viability

#### Evaluation of Cell Viability on HDF Cells

**Figure 11:**
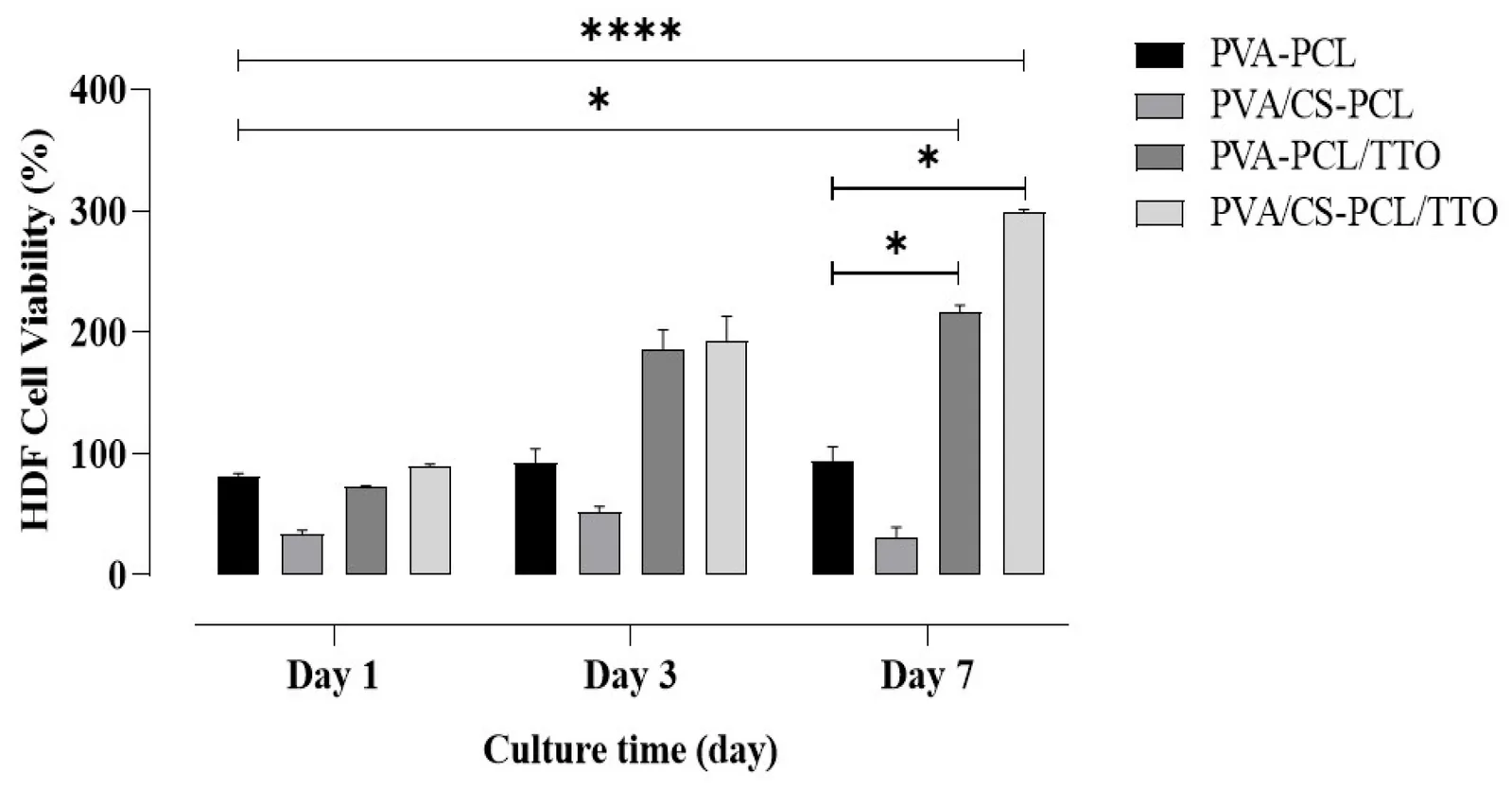
MTT-based metabolic activity of HDF cells cultured with the developed nanofibrous formulations on days 1, 3, and 7. PVA-PCL, which contained neither CS nor TTO, was used as the reference control for comparison with the other formulations. All experiments were performed in triplicate, and the results are expressed as mean ± SEM (*n* = 3) (Abbreviations: PVA-PCL, PVA/CS-PCL, PVA-PCL/TTO, and PVA/CS-PCL/TTO).Statistical significance was determined using one-way ANOVA followed by Tukey’s multiple comparisons test (* *p* < 0.05, **** *p* < 0.0001).

The cellular response to the developed nanofibrous formulations was evaluated by monitoring HDF metabolic activity on days 1, 3, and 7 using the MTT assay (Figure 11). The PVA-PCL formulation, which did not contain CS or TTO, was used as the reference control for comparison with the CS- and/or TTO-containing formulations. Because MTT reduction primarily reflects cellular metabolic activity rather than directly measuring cell number or DNA synthesis, the results were interpreted as an indicator of cellular metabolic activity and cytocompatibility rather than as direct evidence of cell proliferation.

The MTT results showed formulation- and time-dependent differences in HDF metabolic activity relative to the PVA-PCL control. Notably, the PVA/CS-PCL group exhibited a comparatively lower MTT response, indicating that the addition of CS alone did not enhance the cellular metabolic response under the tested conditions. In contrast, the TTO-containing formulations showed higher MTT responses at later time points, with the PVA/CS-PCL/TTO formulation exhibiting the highest metabolic response on Day 7. These findings indicate that TTO incorporation, particularly in combination with CS, was associated with a more favorable HDF metabolic response under the investigated in vitro conditions. However, because the MTT assay does not distinguish changes in cellular metabolic activity from changes in cell number, these findings should not be interpreted as direct evidence of enhanced cell proliferation.

In the complex process of wound healing, fibroblast migration and proliferation contribute to extracellular matrix formation and tissue granulation. Nanofiber-based wound dressings have been widely investigated because their three-dimensional fibrous architecture can provide a microenvironment favorable for cell–material interactions [41]. In the present study, however, these wound-healing processes were not directly evaluated; therefore, the MTT findings are interpreted specifically in terms of HDF metabolic response and cytocompatibility.

The physicochemical characteristics of nanofibrous materials, including surface wettability and fiber morphology, may influence cell–material interactions. In the present study, TTO incorporation decreased the static water contact angle, while SEM analysis demonstrated submicron fibrous morphologies. These material characteristics may contribute to the cellular responses observed in the MTT assay. Nevertheless, specific mechanisms involving cell adhesion, integrin signaling, or mitotic activity were not directly investigated and therefore cannot be established from the present data.

TTO has previously been reported to exhibit antimicrobial and other biological activities when incorporated into electrospun polymeric systems [13]. Similarly, TTO-containing chitosan/PVA-based electrospun materials have been investigated as potential anti-infection wound-dressing platforms [15]. These previously reported properties provide relevant context for the incorporation of TTO into the present hybrid system; however, antimicrobial activity was not directly assessed in this study. Accordingly, the present findings should not be interpreted as experimental evidence of antimicrobial protection.

Previous studies have also described wound-related biological effects of TTO-containing systems, including effects associated with tissue repair and fibrosis-related pathways [41,42]. In particular, reported modulation of α-smooth muscle actin (α-SMA) provides a rationale for further investigation of TTO-containing materials in the context of scar formation [42]. However, neither wound closure nor fibrosis-related biomarkers were evaluated in the present study. Therefore, potential wound-healing and anti-scarring effects of the developed PVA/CS-PCL/TTO platform remain to be established through dedicated migration or wound-closure assays, proliferation-specific analyses, antimicrobial testing, fibrosis-related molecular analyses, and appropriate in vivo wound models.

## 4. Conclusions

In this study, PVA-PCL, PVA/CS-PCL, PVA-PCL/TTO, and PVA/CS-PCL/TTO hybrid nanofibrous mats intended for wound dressing applications were successfully produced using a simultaneous dual-nozzle co-electrospinning method. This novel multi-nozzle configuration successfully eliminated issues such as phase separation and thermodynamic incompatibility commonly encountered in direct blending methods by combining hydrophilic and hydrophobic phases as independent fiber populations within the same matrix. All produced mats exhibited a bead-free, continuous, and homogeneous fiber morphology.

Experimental findings revealed a significant synergy created by CS and TTO regarding the physical, thermal, and mechanical properties of the matrix. The incorporation of hydrophobic TTO markedly improved the surface wettability of the mats while rendering their swelling behavior more balanced and controlled. Meanwhile, CS preserved the matrix’s structural integrity and stabilized mass loss in aqueous environments by forming a strong physical network with the rapidly water-soluble PVA phase. The most remarkable synergy was observed in mechanical strength; the PVA/CS-PCL/TTO mat containing both CS and TTO exhibited high tensile strength and enhanced flexibility, thereby amply meeting the requirements for a flexible yet durable wound dressing.

The in vitro MTT assessment revealed formulation- and time-dependent differences in HDF metabolic activity, with the PVA/CS-PCL/TTO formulation showing the highest metabolic response on Day 7. These findings provide preliminary evidence of a favorable cellular response to the combined CS- and TTO-containing hybrid platform under the investigated in vitro conditions. However, because the MTT assay reflects cellular metabolic activity and does not directly quantify cell proliferation, the present findings should not be interpreted as evidence of enhanced fibroblast proliferation. Moreover, antimicrobial activity, wound closure, tissue regeneration, and scar modulation were not directly investigated in this study. Further studies incorporating antimicrobial assays, proliferation- and migration-specific analyses, fibrosis-related molecular evaluations, and relevant in vivo wound models are therefore required to establish the biological efficacy and wound-healing potential of the developed nanofibrous platform.

## Figures and Tables

**Table 1:** Final composition of the individual spinning phases used to prepare the hybrid nanofibrous mats.

Groups	PVA (% *w*/*v*)	CS (% *w*/*v*)	PVA:CS Solution Ratio (*v*/*v*)	PCL (% *w*/*v*)	TTO (% *v*/*v*)	Tween 80 (% *v*/*v*)
PVA–PCL	13	—	—	10	—	3
PVA/CS–PCL	9.1	0.9	7:3	10	—	3
PVA–PCL/TTO	13	—	—	10	10	3
PVA/CS–PCL/TTO	9.1	0.9	7:3	10	10	3

—: not added.

**Table 2:** Thermal transition temperatures and phase characteristics of hybrid nanofibrous mats derived from DSC analysis.

Groups	Observed Thermal Transitions (°C)	Associated Phase/Event	Thermal Characteristics & Profile Summary
PVA-PCL	59.02 (T_m_)	PCL crystalline phase	Distinct endothermic PCL melting peak (T_m_); stable baseline in the upper temperature range.
PVA/CS-PCL	165.00–175.00 (exothermic), 205.48 (endothermic)	PVA/CS fiber phase	Thermally induced chain reorganization followed by an endothermic transition of the hydrophilic matrix.
PVA-PCL/TTO	212.69 (T*_peak_*)	PVA-rich fiber phase	Endothermic transition characteristic of the electrospun hydrophilic PVA matrix.
PVA/CS-PCL/TTO	59.43 (T_m_) Broad high-T profile	PCL phase & PVA/CS/TTO phase	Retained PCL melting endotherm (T_m_); broadened high-temperature profile influenced by CS/TTO interactions.

**Table 3:** TTO release kinetic parameters of PVA-PCL/TTO and PVA/CS-PCL/TTO.

Model		PVA-PCL/TTO	PVA/CS-PCL/TTO
First-Order	*k*_1_	0.1190	0.1266
	*SE*	0.0224	0.0239
	*R* ^2^	0.9957	0.9952
Higuchi	*k_h_*	0.1716	0.1743
	SE	0.0976	0.1048
	*R* ^2^	0.9199	0.9085
Korsmeyer–Peppas	*k_p_*	0.2137	0.2256
	*n*	0.4268	0.4137
	SE	0.0908	0.0938
	*R* ^2^	0.9370	0.9333
Peppas–Sahlin	*k_d_*	0.1621	0.17441
	*k_r_*	−0.0064	−0.0074
	*m*	0.6994	0.6833
	SE	0.0418	0.0438
	*R* ^2^	0.9880	0.9869

**Table 4:** Water contact angle values of PVA-PCL-based electrospun nanofibrous formulations. Results are expressed as mean ± standard deviation (SD) (*n* = 4).

Sample	Water Contact Angle (°)	Surface Character	Water Contact Angle Images
PVA-PCL	74.78 ± 3.16	Hydrophilic/wettable	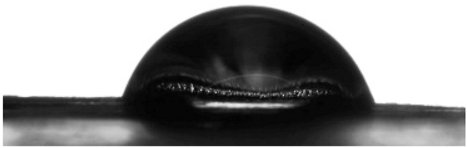
PVA/CS-PCL	74.85 ± 3.64	Hydrophilic/wettable	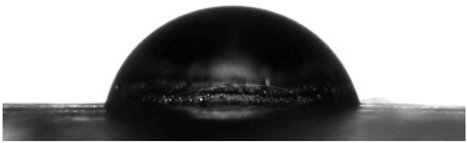
PVA-PCL/TTO	46.69 ± 2.80	More hydrophilic/highly wettable	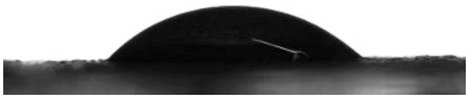
PVA/CS-PCL/TTO	57.05 ± 1.40	Hydrophilic/wettable	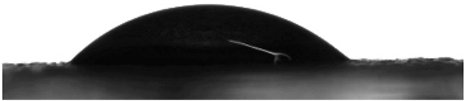

## Data Availability

The original contributions presented in the study are included in the article; further inquiries can be directed to the corresponding authors.

## Supplementary Materials

The following supporting information can be downloaded at https://www.mdpi.com/article/10.3390/polym18202558/s1. Figure S1: Calibration curve of TTO.
