# Supplementary material for: Fabrication and Characterization of Chitosan-Integrated Polyvinyl Alcohol–Polycaprolactone/Tea Tree Oil Nanofibrous Scaffolds via Dual-Nozzle Electrospinning for Wound Dressing Applications
Source: Polymers (Basel). 2026 Oct 17;18(20):2558. doi: 10.3390/polym18202558 (PMC13708919; doi:10.3390/polym18202558)
Supplement: Supplementary file 1 [file polymers-18-02558-s001.zip › polymers-4603569-supplementary.pdf]

polymers

Fabrication and Characterization of Chitosan-Integrated Polyvinyl Alcohol-Polycaprolactone/Tea Tree Oil Nanofibrous Scaffolds via Dual-Nozzle Electrospinning for Wound Dressing Applications

Natavan Ismayilova [1], Tubanur Avci [2], Zekiye Akdag [2], Ridvan Yildirim [2], Zehra Kanli [3], Ahmet Akif Kızılkurtlu [4,5,*], Oguzhan Gunduz [2] and Canan Dogan [2]

1 Chemical Engineering Department, Engineering Faculty, Istanbul University Cerrahpasa, Istanbul 34320, Turkey
2 Department of Metallurgical and Materials Engineering, Faculty of Technology, Marmara University, Istanbul 34722, Turkey
3 Department of Biophysics, Faculty of Medicine, Istanbul Arel University, Istanbul 34010, Turkey
4 Department of Biomedical Engineering, Faculty of Engineering and Natural Sciences, Istanbul Atlas University, Istanbul 34403, Turkey
5 Atlas University Research Center (ARC), Istanbul Atlas University, Istanbul 34403, Turkey
* Correspondence: ahmet.kizilkurtlu@atlas.edu.tr

SUPPLEMENTARY INFORMATIONS (SI)

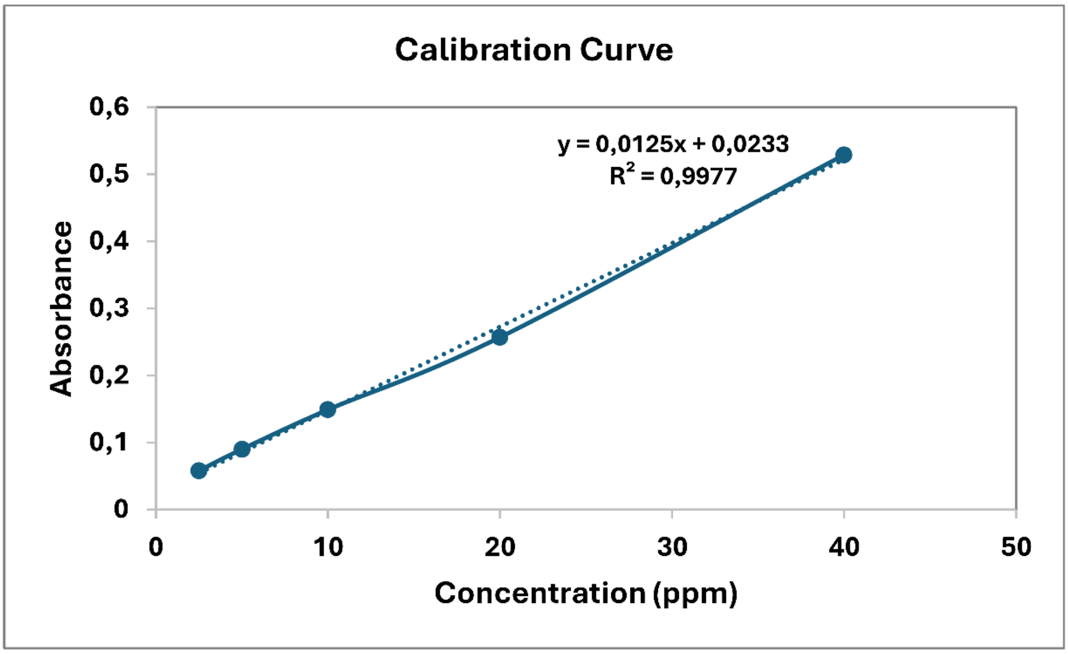

Figure S1: Calibration curve of TTO
